# HBOT as a Potential Adjunctive Therapy for Wound Healing in Dental Surgery—A Narrative Review

**DOI:** 10.3390/jcm15020605

**Published:** 2026-01-12

**Authors:** Beata Wiśniewska, Kosma Piekarski, Sandra Spychała, Ewelina Golusińska-Kardach, Bartłomiej Perek, Marzena Liliana Wyganowska

**Affiliations:** 1 Department of Oral Surgery, Periodontology and Oral Mucosa Diseases, Poznan University of Medical Sciences, 10 Fredry Street, 61-701 Poznan, Poland; ekardach@ump.edu.pl (E.G.-K.); marzenawyganowska@periona.pl (M.L.W.); 2 Arbor Vitae Society of Ethics in Medicine, 103 Marymoncka Street, 01-813 Warsaw, Poland; piekarskikosma@gmail.com (K.P.); sandra.spychalas@gmail.com (S.S.); 3 Department of Cardiac Surgery and Transplantology, Poznan University of Medical Sciences, 61-701 Poznan, Poland; bperek@ump.edu.pl

**Keywords:** hyperbaric oxygen therapy (HBOT), osteoradionecrosis (ORN), medication-related osteonecrosis of the jaw (MRONJ), adjunctive therapy, wound healing, oral surgery

## Abstract

**Background**: Hyperbaric oxygen therapy (HBOT) is considered a potential adjunctive modality to enhance tissue regeneration in oral and maxillofacial surgery. By increasing tissue oxygen availability, HBOT may support bone and soft-tissue repair under hypoxic and chronically inflamed conditions. **Aim**: This narrative review evaluates current experimental and clinical evidence regarding HBOT in high-risk dental indications, including osteoradionecrosis (ORN), medication-related osteonecrosis of the jaw (MRONJ), chronic osteomyelitis, poorly healing postoperative wounds, and procedures in patients with systemic comorbidities. **Methods**: A structured search of PubMed, Web of Science, and the Cochrane Library identified 123 relevant English-language publications (from 1 January 2000–September 2025) addressing HBOT mechanisms and clinical applications in oral and maxillofacial surgery, including clinical trials, observational studies, preclinical models, and systematic reviews. **Results**: Available evidence suggests that HBOT may improve healing outcomes and reduce complication rates in early-stage ORN and MRONJ when used as an adjunct to surgery and systemic therapy. However, findings in implantology—particularly in irradiated or diabetic patients—and in periodontal therapy remain limited, heterogeneous, and methodologically inconsistent. **Conclusions**: HBOT may be considered in selected clinical scenarios, particularly where healing is impaired by hypoxia or systemic disease. Nevertheless, current evidence remains insufficient to support routine use. Standardized, high-quality studies with clearly defined endpoints and uniform therapeutic protocols are needed to determine its clinical effectiveness and optimal indications.

## 1. Introduction

In both soft and hard tissues, the wound-healing process is a multiphase, biologically complex mechanism that includes integrated phases of hemostasis, inflammatory response, cellular proliferation, and tissue remodeling. Under physiological conditions, these stages restore the structural and functional integrity of the damaged site [1]. Their course is regulated by cytokines, local growth factors, and environmental signals such as oxygen availability, immune cell presence, and extracellular matrix composition [2].

The wound-healing process may be disrupted by local or systemic factors. Among the local factors most frequently reported are trauma, local infections (including biofilm-driven contamination), and surgical interventions; importantly, the clinical course depends on the type and dose of the injurious stimulus and on the local and systemic oxidative–antioxidant status (redox balance) [2,3,4,5]. At the systemic level, conditions characterized by tissue hypoxia and immunodeficiency—such as diabetes mellitus, malignancy, chronic kidney disease, decompensated heart failure, and chronic respiratory failure—impair repair by causing cellular energy deficit, reduced phagocytic function, and disturbances of adaptive immunity. This milieu sustains heightened activation of lymphocytes and monocyte–macrophage lineages, shifting cytokine signaling toward a pro-inflammatory profile and perpetuating delayed healing [2,3,4,5]. In patients with diabetes mellitus, careful metabolic stabilization is essential prior to HBOT. The therapy should be postponed when blood glucose levels exceed 300 mg/dL or fall below 100 mg/dL, as both uncontrolled hyperglycemia and hypoglycemia may induce metabolic instability, oxidative stress, and impaired oxygen utilization during hyperoxic exposure. This clinical precaution has been emphasized in safety analyses of HBOT and reflects recommendations in current practice guidelines [6,7]. A major clinical challenge arises in wound healing in areas subjected to radiotherapy, especially in patients treated for head and neck cancers [8]. In this context, radiotherapy refers primarily to external beam radiation therapy (teletherapy), which delivers ionizing radiation from an external source through the skin into deeper tissues. This modality causes diffuse, progressive, and often irreversible injury to normal tissues due to endothelial cell loss, capillary rarefaction, fibroblast senescence, and oxidative stress–related hypoxia. These mechanisms lead to impaired angiogenesis, fibroatrophic tissue transformation, and depletion of local progenitor cells, thereby reducing regenerative capacity [8,9]. In contrast, brachytherapy (contact irradiation), where radioactive sources are placed directly within or adjacent to the tumor, produces more localized effects with sharper dose gradients and less extensive hypoxia in surrounding tissues. Nevertheless, both modalities can impair wound healing by promoting chronic inflammation, microvascular thrombosis, and persistent oxidative stress [10,11].

Histological and immunohistochemical studies demonstrate a reduced number of blood vessels identified by endothelial markers CD31+ and CD34+, as well as increased expression of profibrotic mediators such as TGF-β1 and CTGF, which promote fibrosis and perpetuate chronic tissue damage [9]. These disturbances are particularly relevant in the oral cavity, where proper healing of soft and hard tissues depends on adequate vascularization, oxygen availability, and the presence of active repair cells. In irradiated patients, these resources are significantly diminished, leading to delayed post-extraction wound healing, risk of dehiscence, infection, and development of necrotic lesions, including osteoradionecrosis [12,13,14]. The reduced angiogenic potential, combined with oxidative stress and impaired immune regulation, gradually leads to loss of physiological tissue homeostasis following radiation exposure. In this altered microenvironment, even minor injuries such as tooth extractions, implant procedures, or small surgical interventions can trigger a cascade of chronic and clinically challenging complications. Management of such sequelae often requires adjunctive regenerative therapies, including HBOT or treatments based on local growth factors aimed at restoring favorable healing conditions [15,16].

In some patients, ORN develops, defined as mandibular or maxillary bone necrosis within a previously irradiated field, presenting as a non-healing area of exposed bone persisting for at least three months in the absence of tumor recurrence [17]. The pathogenesis of ORN is primarily driven by chronic hypoxia, ischemia, loss of regenerative cells, and tissue fibrosis. These factors compromise local repair mechanisms, and in the presence of surgical trauma or infection, further destabilize tissue integrity, especially in the maxilla and mandible. This environment leads to chronic necrotic changes and necessitates supportive regenerative therapies [8,17,18]. In response to wound healing difficulties in hypoxic tissues, hyperbaric oxygen therapy has been proposed. Typical clinical HBOT protocols use 100% oxygen at 2.0–2.8 ATA for 60–120 min per session, usually administered once daily for 20–40 sessions in chronic wound or osteoradionecrosis protocols [19,20]. For post-radiation necrosis, many protocols prescribe 2.4 ATA for 80–90 min with 30 pre- and 10 post-operative sessions, as used by Annane et al. in a placebo-controlled trial [21]. Comparative data show similar outcomes between 2.0 ATA/120 min and 2.4 ATA/90 min regimens in radiation cystitis, suggesting therapeutic equivalence with fewer sessions at higher pressure [22]. Safety guidelines recommend intermittent air breaks during longer exposures to prevent oxygen toxicity [6]. The therapy promotes regeneration by stimulating angiogenesis, activating fibroblasts, enhancing collagen synthesis, and reducing chronic inflammation. These effects have been confirmed in Ortega’s review on the molecular mechanisms of HBOT and in De Wolde’s systematic analysis focusing on oxidative stress, angiogenesis, and inflammation [23,24]. Available literature on HBOT’s impact on wound healing in dentistry, with particular emphasis on ORN, remains limited, and published studies and meta-analyses present diverse and often contradictory results. A critical evaluation of HBOT’s effectiveness in dental clinical settings is therefore still required. In the review by Bennett et al., the authors concluded that the quality of evidence supporting HBOT in chronic wound management, including ORN, remains low to moderate, and the need for further well-designed studies is evident [19]. The aim of the present study is to provide a narrative review of the literature analyzing the role of hyperbaric oxygen therapy in oral surgery, with reference to its impact on wound healing.

## 2. Materials and Methods

### 2.1. Study Design and Objectives

This narrative review synthesizes available evidence on HBOT in oral and maxillofacial surgery. It emphasizes wound-healing indications such as ORN, MRONJ, healing after extractions and implant placement, and periodontal regenerative procedures, while also addressing uncommon jaw conditions such as osteopetrosis-associated osteomyelitis, primary chronic osteomyelitis in children, and dysplastic lesions (cf. Section 5.5).

### 2.2. Literature Search

Searches were conducted in PubMed/Medline (National Library of Medicine, Bethesda, MD, USA), Web of Science (Clarivate Analytics, Philadelphia, PA, USA), Scopus (Elsevier, Amsterdam, The Netherlands), and the Cochrane Library (Cochrane Collaboration, London, UK) for English-language publications from 1 January 2000 to 20 September 2025. The strategy combined Medical Subject Headings (MeSH) and free-text terms covering HBOT (hyperbaric oxygenation, hyperbaric oxygen therapy, HBOT, HBO) and indication clusters relevant to oral and maxillofacial surgery (osteoradionecrosis, MRONJ, dental implants and tooth extractions, osseointegration, periodontitis/periodontal therapy, regenerative surgery and tissue regeneration, osteomyelitis—including primary chronic, nonbacterial and pediatric forms—osteopetrosis, and cemento-osseous dysplasia). Syntax was adapted to each database (e.g., topic search in Web of Science, Title/Abstract keywords in PubMed). Publisher platforms (e.g., ScienceDirect—Elsevier, Amsterdam, The Netherlands) were used to obtain full texts and to run targeted, topic-focused searches within Elsevier journals using the same keywords; records were screened alongside database outputs and de-duplicated prior to inclusion. All included publications were peer-reviewed; non-indexed or non-peer-reviewed materials were excluded to minimize selection bias.

### 2.3. Eligibility and Selection

Titles and abstracts were screened by the authors for relevance to HBOT in oral and maxillofacial indications; full texts of potentially relevant records were assessed against eligibility criteria aligned with the review objectives. Eligible studies included randomized and non-randomized clinical trials, prospective and retrospective observational studies, case series, case reports, and systematic reviews or meta-analyses examining HBOT in any of the above clinical contexts. Preclinical (in vivo and in vitro) studies were considered when they elucidated mechanisms pertinent to tissue repair, angiogenesis, oxidative/inflammatory modulation or osseointegration. Excluded were non-peer-reviewed items, conference abstracts without analyzable data, non-English publications, and studies unrelated to HBOT or dental/maxillofacial indications.

### 2.4. Data Extraction and Synthesis

After automatic and manual de-duplication, 899 unique records remained across sources; 123 publications were retained for qualitative (narrative) synthesis following full-text assessment. Findings were summarized qualitatively and organized by clinical domain (osteoradionecrosis, MRONJ, implantology, periodontology, and rare maxillomandibular conditions). Key outcomes were compared narratively with reference to study design, HBOT parameters, co-interventions, and reported benefits or limitations. Given the heterogeneity of study designs, indications, and HBOT protocols, evidence was synthesized narratively without statistical pooling.

## 3. Physiological Basis of Hyperbaric Oxygen Therapy

HBOT involves administering 100% oxygen to the patient under increased ambient pressure in a hyperbaric chamber. Standard therapeutic pressures range between 2 and 3 atmospheres absolute (ATA), resulting in a significant rise in partial oxygen pressure (ppO_2_) and plasma oxygen solubility. This enables tissue oxygenation independently of hemoglobin, even in regions with compromised perfusion or hypoxia [23]. Clinical HBOT protocols vary depending on the indication, treatment goal, and tissue sensitivity to oxygen exposure. Most therapeutic regimens employ pressures between 2.0 and 3.0 ATA, corresponding to inspired pO_2_ of approximately 1520–2280 mmHg, with individual sessions lasting 60–120 min. Treatments are typically administered once daily, 5–7 times per week, over a total of 20–40 sessions for chronic wounds or osteoradionecrosis, whereas acute conditions such as carbon monoxide poisoning or air embolism may require shorter, high-pressure courses at 2.8–3.0 ATA [19,20,25]. Despite the established physiological rationale, the heterogeneity of treatment protocols—involving different pressures, exposure times, and total number of sessions—remains a major limitation when interpreting clinical efficacy across medical fields. Comparative studies have reported variable outcomes for 2.0 ATA versus 2.4 ATA exposures in radiation-induced injury and wound healing, with similar efficacy but differences in duration and frequency requirements [6,22]. This diversity in methodology contributes to the ongoing debate regarding HBOT’s clinical relevance and underlines the need for standardized dosing protocols supported by controlled studies.

According to Henry’s law, increased pressure elevates the amount of oxygen dissolved in plasma, thereby facilitating its diffusion into hypoxic tissues. At 2.0 ATA, plasma oxygen concentration may reach 4–6 mL/dL, and at 2.0–2.5 ATA, a tenfold increase in physically dissolved oxygen can be observed, generating a favorable diffusion gradient into ischemic regions [6,26]. At the molecular level, HBOT induces a transient increase in oxidative stress through the generation of reactive oxygen and nitrogen species (ROS/RNS), which act as signaling mediators. This effect is modulated via the NF-κB/IκBα pathway, leading to reduced pro-inflammatory cytokine levels and attenuation of inflammatory activity [24]. Moreover, alternating exposure to hyperoxia and return to normoxia triggers transient redox changes that may secondarily stabilize HIF-1α and stimulate VEGF expression along with other pro-angiogenic factors—a mechanism described as intermittent hyperoxia [26,27]. HBOT also exerts beneficial effects on immune function. Enhanced oxygen availability allows neutrophils to generate ROS more effectively, strengthening antimicrobial activity. Additionally, the therapy modulates inflammatory responses by suppressing NF-κB and reducing pro-inflammatory cytokines, thereby facilitating the transition of tissues into the proliferative repair phase [28,29,30]. Hyperbaric oxygen also influences microcirculation. Oxygen-induced vasoconstriction reduces vascular permeability and tissue edema, while simultaneously improving perfusion and supporting regenerative processes [7,31]. Despite its therapeutic benefits, excessive exposure to high oxygen pressures may result in toxicity, particularly within the central nervous system at pO_2_ ≥ 1.6 ATA. This clinical limitation necessitates the use of a restricted number of sessions and incorporation of air breaks during treatment to mitigate the risk of complications [32,33]. Experimental and clinical data indicate that the risk–benefit balance of oxygen exposure depends on both partial pressure and exposure time. Short normobaric hyperoxia at approximately 30–40% O_2_ can be used without overt pulmonary or CNS toxicity in selected settings; however, even within this range, transient increases in oxidative markers have been documented in humans after 60 min of exposure [34,35]. At higher inspired fractions or with prolonged exposures, excessive formation of reactive oxygen and nitrogen species is accompanied by inflammatory cell recruitment and tissue injury; in vitro, even 40% O_2_ can induce senescence-like changes in human fibroblasts, underscoring the role of duration and model [36]. In the hyperbaric setting, elevated ppO_2_ increases the risk of CNS and pulmonary oxygen toxicity; clinical practice therefore employs pressure/time limits and scheduled air breaks to mitigate risk [37,38].

In summary, HBOT exerts its physiological effects by increasing tissue oxygen availability, modulating redox signaling pathways, and regulating vascular and immune responses. These mechanisms collectively support the transition from inflammation to regeneration. Most of the evidence discussed in this section derives from in vitro and in vivo studies, which provide important mechanistic insights into how oxygen exposure influences cell proliferation, angiogenesis, and extracellular matrix formation. The following subsections (Section 3.1.1, Section 3.1.2 and Section 3.1.3) present these findings in greater detail, focusing on the cellular and molecular effects of HBOT—particularly its impact on fibroblast activity, angiogenesis, and collagen synthesis, which form the biological foundation of wound healing.

### 3.1. Effects of HBOT on Fibroblasts, Angiogenesis, and Collagenogenesis

#### 3.1.1. Fibroblast Proliferation and Activity

In vitro experiments showed that fibroblasts cultured in serum-deprived conditions and exposed daily to hyperbaric oxygen therapy (one-hour session) for seven consecutive days at pressures ranging from 1.0 to 3.0 ATA exhibited a heterogeneous proliferative response. While initial exposure to high oxygen concentrations appeared to suppress their mitotic rate, from day seven onwards a marked increase in proliferation was observed at pressures near 2.0 ATA. At the same time, an increase in autocrine secretion of bFGF was noted, whereas a modest elevation in VEGF levels was detected after the very first therapeutic session [39]. The conversion of a local inflammatory environment into one favorable for regeneration is further supported by enhanced fibroblast migration, as documented in experimental models using mice with diabetic skin wounds. HBOT activates the HIF-1α signaling pathway and increases the expression of key proangiogenic factors such as VEGF and SDF-1, along with their receptors VEGFR2 and CXCR4. These molecular changes promote accelerated fibroblast proliferation and intensified migration of both endothelial cells and fibroblasts toward damaged sites requiring repair. The result of this multifactorial process is enhanced granulation tissue formation and accelerated wound healing, even under metabolic dysregulation characteristic of chronic hyperglycemia [40,41,42].

In summary, in vitro and in vivo evidence indicates that HBOT enhances fibroblast proliferation, migration, and growth factor secretion, thereby creating a regenerative microenvironment that promotes effective granulation tissue formation and accelerates wound healing.

#### 3.1.2. Angiogenesis and Vasculogenesis

Neovascularization represents one of the principal therapeutic targets of HBOT. The therapy increases oxygen availability in tissues, directly stimulating VEGF expression and supporting mobilization of endothelial progenitor cells (EPCs), which play a central role in neovascularization. Clinical studies have confirmed HBOT-induced angiogenesis and improved tissue perfusion under ischemic-inflammatory conditions [24]. Similar findings have been validated in in vivo models, where HBOT accelerated EPC trafficking from the bone marrow to sites of injury and induced significant upregulation of VEGF and angiogenesis markers [43]. Furthermore, a literature review on HBOT mechanisms highlighted the synergistic interplay of angiogenesis and vasculogenesis, modulated by reactive oxygen species (ROS), nitric oxide (NO), and lactate, collectively accelerating vascular network restoration [44]. NO plays a key regulatory role in HBOT-induced angiogenesis through activation of nitric oxide synthase (NOS) pathways. Hyperoxic exposure transiently enhances endothelial and neuronal NOS (eNOS and nNOS) activity, increasing NO generation and promoting vasodilation, endothelial migration, and proliferation, which collectively enhance vascular repair. In contrast, sustained or excessive induction of inducible NOS (iNOS) under prolonged or high-pressure conditions may augment reactive nitrogen species (RNS) formation and inflammation. Clinical and translational studies consistently support these mechanisms. HBOT significantly increases wound-fluid NO metabolites (NOx) in patients with chronic wounds, confirming activation of NO-dependent repair signaling [45]. Controlled human trials demonstrate that exposure to 100% O_2_ at 2.5 ATA significantly elevates plasma and exhaled NO, reflecting enhanced vascular NOS activity [46]. Experimental evidence further shows that HBOT promotes vasculogenic stem-cell mobilization and neovascularization through NO- and ROS-dependent signaling involving eNOS and VEGF activation [44].

In summary, evidence from in vivo, translational, and clinical studies demonstrates that HBOT promotes angiogenesis and vasculogenesis primarily through NO- and ROS-dependent signaling and balanced activation of eNOS/nNOS pathways, while excessive iNOS induction may counteract these benefits by increasing oxidative and inflammatory stress.

#### 3.1.3. Collagen Synthesis and Extracellular Matrix Organization

During wound healing, type III collagen is among the earliest extracellular matrix components synthesized. Animal model studies demonstrated that HBOT enhances type III collagen production in the early healing phase, supporting the development of a stable tissue scaffold while simultaneously limiting excessive vascularization at this stage, thereby promoting better matrix organization [47,48]. Moreover, HBOT has been shown to significantly improve skin structure in aging models by increasing collagen and elastin fiber density and reducing the number of senescent keratinocytes, indicating a rejuvenating potential of oxygen-induced hyperoxia [49]. Table 1 summarizes the key mechanisms of HBOT, describing their biological basis, main therapeutic effects, and literature sources confirming the role of hyperoxia in modulating perfusion, oxidative stress, angiogenesis, fibroblast activity, and immune responses.

In conclusion, the physiological basis of HBOT provides a coherent mechanistic framework explaining its potential efficacy in conditions involving hypoxia and impaired tissue repair. By enhancing oxygen availability and modulating redox-sensitive signaling pathways, HBOT promotes fibroblast proliferation, angiogenesis, and collagen matrix organization—key processes underpinning effective tissue regeneration. Experimental findings from in vitro and in vivo models consistently demonstrate these beneficial effects; however, translational limitations remain, as variations in pressure, exposure time, and biological context may not fully reflect clinical outcomes. These mechanistic insights nevertheless form the foundation for the following sections, which explore the therapeutic implications of HBOT in oral and maxillofacial surgery, including ORN and MRONJ, considering current evidence, challenges, and future research directions.

## 4. Applications of HBOT in General Medicine as a Reference Point

HBOT is increasingly applied as an adjunctive therapeutic method to support healing of chronic non-healing wounds, which provides a rationale for its potential implementation as supportive therapy in oral surgery, particularly in cases of chronic osteomyelitis or postoperative inflammatory complications. Numerous clinical studies and meta-analyses document its efficacy in various contexts.

A 2013 meta-analysis including six randomized clinical trials and six observational studies in patients with non-healing lower limb ulcers associated with diabetes reported that HBOT reduced the risk of major amputations by 12% in observational studies and by 24% in RCTs [50]. Despite methodological limitations, these findings highlight the potential value of HBOT as supportive therapy in diabetic patients with impaired wound healing. Similarly, a systematic review published by Löndahl in 2013 emphasized that although HBOT had previously been considered poorly evidenced, two well-designed randomized, double-blind, placebo-controlled trials significantly improved the quality of evidence supporting its efficacy in chronic diabetic foot ulcer healing, thereby identifying a specific patient subgroup who may benefit from HBOT [51]. A systematic review and meta-analysis published in 2021 in Scientific Reports included fourteen clinical studies—twelve RCTs and two non-randomized controlled trials—with a total of 768 participants. The authors demonstrated that HBOT provides significant clinical benefits in patients with diabetic foot ulcers. HBOT use was associated with a markedly higher rate of complete ulcer healing (odds ratio 0.29; 95% CI: 0.14–0.61), corresponding to a significant reduction in the risk of non-healing compared with controls. Moreover, a significant reduction in major amputations was observed (RR = 0.60; 95% CI: 0.39–0.92), whereas the impact on minor amputations was not statistically significant (RR = 0.82; 95% CI: 0.34–1.97) [52]. Conversely, a 2020 meta-analysis published in the Journal of Vascular Surgery, focusing on patients with diabetic foot ulcers coexisting with peripheral arterial occlusive disease (PAOD), indicated limited efficacy of HBOT in selected clinical outcomes. The analysis included eleven studies with 729 participants and found that while HBOT significantly reduced the risk of major amputations (from 26% to 10.7%, *p* = 0.002; NNT = 7), its effect on other therapeutic outcomes was inconsistent. No statistically significant differences were reported in the frequency of minor amputations (RR = 0.82; 95% CI: −13% to +30%) or in complete wound healing, with three trials yielding heterogeneous and inconclusive results. The authors concluded that HBOT effectiveness in ischemic DFU may be confined to reducing the risk of severe complications such as major amputations, but not necessarily to accelerating wound healing or reducing the need for minor interventions [53].

In the management of chronic osteomyelitis, an increasing body of systematic review evidence suggests a beneficial effect of HBOT when used in addition to standard surgical and antibiotic treatment. One of the most comprehensive reviews, published in 2018 by Savvidou et al., included 45 studies involving 460 patients with chronic osteomyelitis. The review found that HBOT achieved sustained remission without infection recurrence in 308 patients, corresponding to a success rate of 73.5%. This high rate of favorable outcomes underscores the potential of HBOT as a valuable adjunct in refractory osteomyelitis, particularly in cases where conventional treatment fails or recurrence risk is high [54]. Retrospective studies also confirm HBOT efficacy in osteomyelitis. In one such study involving patients with acute femoral osteomyelitis, complete recovery was reported in 12 of 13 patients (92%) without treatment-related complications [55]. These findings suggest HBOT may be particularly useful as supportive therapy in difficult-to-treat bone infections. The mechanism of HBOT in this context is well documented, with increased oxygen partial pressure in affected tissues promoting angiogenesis, improving perfusion, and enhancing leukocyte defense functions—particularly phagocytosis and bactericidal activity—leading to more effective pathogen clearance. These effects act synergistically with surgery and antibiotics, supporting bone regeneration and preventing recurrence of chronic inflammation [56].

Clinical practice also provides examples where HBOT achieves significant, sometimes breakthrough outcomes, particularly when standard therapy fails. A case published in the Annals of Thoracic Surgery described refractory sternal osteomyelitis following sternotomy, which was successfully treated with HBOT despite lack of response to conventional methods [57]. Another notable case, reported by Kjellberg et al. (2024), described successful HBOT treatment in a child with a rare genetic disease complicated by pseudoarthrosis [58]. Although partly related to the immunomodulatory effects of HBOT in ARDS following COVID-19, completion of the healing process after 40 HBOT sessions highlights its potential efficacy in rare, treatment-resistant conditions [58]. From a broader perspective, the clinical guidelines issued by the Undersea & Hyperbaric Medical Society (UHMS) in 2015 formally recommend HBOT as an adjunctive therapy in Wagner grade 3 or higher diabetic foot ulcers. HBOT should be considered in patients showing no improvement after at least 30 days of standard conservative management. UHMS emphasizes the moderate quality of evidence and the importance of shared decision-making with patients [59]. Although many systematic reviews emphasize methodological limitations of HBOT studies, available meta-analyses and reviews consistently indicate clinical benefits. Evidence suggests HBOT may accelerate wound healing, reduce the number of major amputations in diabetic patients, aid in controlling chronic bone infections, and provide effective supportive therapy in cases resistant to standard treatment [53,60,61].

From the perspective of oral surgery, the multifaceted mechanisms of HBOT gain particular importance, especially in high-risk cases such as jaw osteonecrosis, chronic osteomyelitis, or poorly healing postoperative wounds. HBOT effects—including improved perfusion, angiogenesis stimulation, anti-inflammatory and antibacterial activity—may significantly reduce complication risks and support tissue regeneration within the oral cavity [20,62]. In oral surgical scenarios—such as post-radiotherapy in the head and neck region or in chronic inflammatory conditions—tissues are often hypoxic and poorly vascularized. Increased oxygen availability through HBOT may not only accelerate reparative processes but also enhance the efficacy of antibiotics, acting synergistically with them [21]. The potential benefits of HBOT described in the context of diabetic and ischemic wound management [39,40] may therefore translate into dental practice, particularly in areas such as reducing the risk of osteoradionecrosis [21,63], supporting implant osseointegration in post-radiotherapy patients, or improving wound healing outcomes following surgical procedures in individuals with local or systemic risk factors [64,65].

## 5. HBOT in Oral Surgery: Literature Review

### 5.1. Osteoradionecrosis of the Maxilla and Mandible

ORN of the jaws represents one of the most serious late complications of radiotherapy for head and neck cancers. Its pathophysiology involves radiation-induced endarteritis, progressive fibrosis, and chronic hypoxia, which together impair angiogenesis, bone remodeling, and tissue repair capacity [66].

#### 5.1.1. Evidence from Systematic Reviews and Randomized Trials

Recent high-quality systematic reviews and meta-analyses have shown that the role of HBOT in the prevention and management of ORN remains controversial. The Cochrane Review on Late Radiation Tissue Injury found moderate-quality evidence supporting HBOT in certain soft-tissue radiation injuries (e.g., cystitis, proctitis), but data for osseous complications such as ORN were insufficient and heterogeneous [67]. Similarly, the Cochrane Review on Interventions for Preventing ORN concluded that HBOT did not show a clear prophylactic advantage over standard care in irradiated patients, stressing the small size and low power of available RCTs [68]. The HOPON randomized controlled trial remains the most rigorous prospective study in this field. Among 144 randomized participants undergoing dentoalveolar surgery in irradiated mandibles (>50 Gy), the incidence of ORN was 6.4% in the HBOT group and 5.7% in controls, indicating no statistically significant difference. The authors emphasized that the overall risk of ORN was low in both groups, reflecting improved contemporary radiotherapy techniques and oral surgical care [20].

#### 5.1.2. Observational and Translational Data

Contrary to the neutral RCT results, retrospective and translational data suggest that HBOT may provide clinical benefit in early-stage ORN. A cohort study from Thailand including 84 patients reported enhanced healing in stages I–II, while in stage III the benefit was limited despite additional HBOT sessions [69]. Consistent findings were observed in the systematic review by Mohandas et al., which concluded that HBOT may be useful as an adjunct to conservative surgery and antibiotic therapy, particularly in early or moderate lesions, though overall evidence quality was low-to-moderate due to small, heterogeneous studies [70]. A recent meta-analysis comparing treatment strategies in mandibular ORN demonstrated that combined HBOT + surgery achieved higher rates of lesion resolution than either therapy alone, albeit with substantial methodological variability [71]. In contrast, state-of-the-art reviews highlight a paradigm shift away from routine HBOT, emphasizing the growing role of medical management (e.g., pentoxifylline-tocopherol-clodronate, “PENTO/PENTOCLO”) and precision surgery [72,73]. The ASCO–ISOO–MASCC Clinical Practice Guideline (2024) concludes that HBOT should not be used routinely for the prevention or treatment of ORN, but may be considered on a case-by-case basis in early-stage disease or as an adjunct to surgery in centers with appropriate expertise [74].

The most relevant clinical studies, systematic reviews, and guidelines evaluating the role of HBOT in the prevention and management of ORN are summarized in Table 2.

#### 5.1.3. Limitations and Future Directions

Despite an extensive body of literature, the current evidence supporting HBOT for ORN is limited by several methodological and clinical factors. The major systematic reviews highlight heterogeneity of study protocols, including variation in pressure (1.8–3.0 ATA), session duration (60–120 min), and total number of exposures (20–40), which complicates direct comparisons and quantitative synthesis [67,68,70]. Moreover, different staging systems and endpoints—such as “complete healing,” “clinical improvement,” or “avoidance of major surgery”—introduce inconsistency in outcome reporting and lower the certainty of meta-analytical conclusions [70,71]. Another key limitation lies in sample size and event frequency. Modern radiotherapy techniques (e.g., IMRT) and improved dental preventive care have substantially reduced the baseline incidence of ORN, making it difficult for contemporary RCTs—such as the HOPON trial—to achieve sufficient statistical power. In HOPON, both HBOT and control groups demonstrated a low ORN rate (<7%), limiting the capacity to detect moderate treatment effects [20]. This issue has been repeatedly emphasized in systematic reviews and guidelines, which call for adequately powered studies specifically designed for high-risk cohorts [67,74]. Another methodological problem arises from mixing preventive and therapeutic indications within reviews and clinical protocols. Preventive studies assess HBOT administered before dentoalveolar procedures in irradiated mandibles, whereas therapeutic studies involve established ORN; merging these fundamentally different populations may obscure any potential benefit [68,72]. Additionally, most retrospective analyses were performed in heterogeneous patient groups receiving variable adjunctive treatments—such as antibiotics, pentoxifylline-tocopherol-clodronate (PENTO/PENTOCLO), or surgical debridement—which introduces significant confounding by co-interventions [70,73,74]. In clinical practice, treatment timing and disease stage appear to be critical determinants of HBOT efficacy. Evidence suggests that the therapy may enhance healing predominantly in early stages (Notani I–II), where hypoxia and inflammation dominate but necrosis remains limited. In contrast, in advanced stage III disease—characterized by pathological fractures and full-thickness bone necrosis—the potential for HBOT to reverse ischemic damage is biologically constrained, and surgical resection remains the mainstay of management [69,71,72]. This stage-dependent response may explain the positive signals observed in early-stage observational data versus the neutral results from large randomized trials. From a mechanistic standpoint, HBOT’s failure to demonstrate consistent benefit in ORN may relate to the irreversible structural damage and fibrosis in chronically irradiated bone. While increased oxygen tension can enhance angiogenesis and fibroblast activity, these effects are insufficient to regenerate devascularized cortical bone with obliterated microcirculation. Additionally, repeated high-pressure exposures may induce oxidative stress, potentially counteracting the intended regenerative signaling effects if not properly titrated [67,70]. Adverse events and logistical barriers further limit the broad application of HBOT. Reported side effects include middle-ear barotrauma and transient myopia, while the therapy itself is resource-intensive and requires strict adherence to multiweek schedules—factors that can affect compliance and real-world feasibility [67,74].

#### 5.1.4. Future Research Priorities

To clarify the therapeutic role of HBOT in ORN, future clinical trials should address key methodological deficiencies identified across current studies. First, RCTs should be designed separately for prophylactic and therapeutic indications, with stratification by validated staging systems (e.g., Notani or Lyons) and standardized surgical co-interventions. Endpoints should focus on clinically meaningful outcomes, such as complete mucosal coverage, time to lesion resolution, avoidance of segmental resection, and patient-reported quality of life [71,74]. Second, there is a pressing need for standardized HBOT protocols, defining optimal pressure (around 2.4 ATA), exposure time (90 min), number of sessions (20–30 pre/post), and use of air breaks. Prospective trials should incorporate adherence monitoring and standardized toxicity reporting according to CTCAE criteria [20,67]. Third, confounding by adjunctive therapies should be minimized through controlled co-treatment protocols—defining antibiotic use, PENTO regimen, and surgical technique—to isolate the independent contribution of HBOT [70,72]. To address low event rates, future studies could focus on high-risk populations (e.g., >60–66 Gy mandibular dose, diabetics, smokers) or adopt adaptive or cluster-randomized designs [68,74]. Finally, translational studies should integrate biomarkers and imaging of tissue perfusion—such as near-infrared spectroscopy or dynamic contrast-enhanced MRI—to identify responders and optimize patient selection [72]. Economic analyses are also warranted to assess cost-effectiveness, as resource utilization remains a critical determinant of clinical adoption [67,74].

In summary, although HBOT has a strong physiological rationale and encouraging preclinical data, current clinical evidence remains inconclusive. The therapy may provide benefit in early, less advanced ORN or as an adjunct to surgery, but routine use is not supported by present RCTs or guidelines [20,70,74]. Future rigorously designed multicenter trials with standardized methodology, precise staging, and modern endpoints are essential to determine the true therapeutic value of HBOT in this setting. Given the overlapping pathophysiological mechanisms of vascular compromise and impaired bone remodeling, many of the lessons learned from ORN may also inform the potential applications of HBOT in medication-related MRONJ.

### 5.2. Medication-Related Osteonecrosis of the Jaw

MRONJ is a complex and challenging complication associated with antiresorptive and antiangiogenic therapies used for osteoporosis and oncologic bone disease. Its multifactorial pathophysiology involves suppression of osteoclastic bone resorption, impaired angiogenesis, chronic inflammation, and reduced bone remodeling leading to ischemic necrosis of the maxillofacial skeleton [75,76]. The therapeutic role of HBOT in MRONJ remains under investigation. The only randomized controlled trial to date, conducted by Freiberger et al., included 49 randomized patients (46 analyzed) with bisphosphonate-related MRONJ. HBOT was administered at 2.0 ATA for 90 min, twice daily, for a total of 40 sessions, as an adjunct to surgical debridement and antibiotic therapy. Clinical improvement was observed in 68% of HBOT-treated patients compared with 38.1% of controls (*p* = 0.043). HBOT significantly shortened the time to clinical improvement (39.7 vs. 67.9 weeks; *p* = 0.03) and accelerated pain resolution (*p* < 0.01), although complete mucosal healing was not statistically different between groups (52% vs. 33.3%; *p* = 0.203). These findings indicate that HBOT provides symptomatic and functional benefit but does not consistently achieve complete osseous healing [77]. Earlier observational evidence from a prospective case series by Freiberger et al. involving 16 patients with bisphosphonate-related osteonecrosis of the jaw (BP-ONJ) reported clinical improvement in 87.5% and long-term remission in 62.5% of cases. Patients who continued bisphosphonate therapy during HBOT experienced significantly earlier recurrence (8.5 vs. 20.1 months; *p* = 0.006). Continuation of bisphosphonate therapy during HBOT was associated with earlier disease recurrence in this prospective series, suggesting that treatment suspension may enhance clinical outcomes [62]. Although the study provided early supportive evidence for the potential adjuvant role of HBOT in early-stage MRONJ, the absence of a control group and the small sample size limit the generalizability of its findings [62]. The Cochrane systematic review by Beth-Tasdogan et al. evaluated all available interventions for MRONJ management, including HBOT, and identified only one randomized controlled trial (Freiberger et al., 2012) [77]. The authors concluded that the overall certainty of evidence for HBOT is very low due to small sample sizes, methodological heterogeneity, and the absence of standardized outcome measures. No clear benefit could be demonstrated beyond potential symptomatic improvement [78]. Similarly, Govaerts et al., in a PRISMA-based systematic review of adjuvant therapies such as laser, platelet-rich fibrin (L-PRF), fluorescence-guided surgery, and HBOT, found that evidence for hyperbaric oxygen was limited to small, heterogeneous case series, and emphasized the need for high-quality randomized multicenter trials [79].

Narrative and integrative reviews have reinforced the uncertain but biologically plausible role of HBOT in MRONJ management. Ceponis et al. described the mechanistic rationale for hyperbaric oxygen—via enhanced angiogenesis, fibroblast proliferation, and modulation of cytokine and immune responses—but emphasized ongoing controversy regarding clinical efficacy, cost-effectiveness, and access. The authors recommended a multimodal approach combining surgery, antibiotic therapy, and HBOT in selected cases of medication- or radiation-related osteonecrosis [80]. Similarly, Biancardi et al. reviewed published case reports and small clinical series, noting improvement in pain and quality of life among patients treated with HBOT, but confirming the absence of controlled evidence or standardized treatment protocols [81]. Emerging clinical evidence, such as the case report by Tanabe et al., describes complete remission of osteoradionecrosis after 20 HBOT sessions at 2.4 ATA, without adverse events—further emphasizing the need for standardized and prospective research protocols [82]. Recent systematic and narrative reviews continue to support the potential but unproven role of HBOT in MRONJ management. Frutuoso et al., in a PRISMA-based systematic review of case reports and series, concluded that HBOT may assist wound healing and symptom relief in selected patients, though evidence remains at a very low level [83]. Similarly, Byrne et al. highlighted HBOT as an optional adjunctive measure within a comprehensive multidisciplinary approach for cancer patients receiving bone-modifying agents [84]. The main clinical trials and systematic reviews assessing the role of HBOT as an adjunctive or supportive treatment in MRONJ are summarized in Table 3.

Despite several reports of symptomatic or partial clinical improvement with HBOT in MRONJ, the overall certainty of evidence remains low. Most studies are constrained by small sample sizes, heterogeneous patient populations, and non-standardized HBOT protocols (e.g., pressures 2.0–2.8 ATA; 20–60 sessions), which limits comparability and precludes robust meta-analysis. HBOT is frequently combined with surgery, antibiotics, or other adjunctive pharmacotherapies, introducing confounding by co-interventions that obscures HBOT’s independent effect [78,79]. A further limitation is the lack of blinding and control groups in most case series and observational studies. The only randomized controlled trial reported earlier pain reduction and symptomatic improvement, but no statistically significant increase in complete mucosal closure [77]. This pattern suggests HBOT may primarily modulate inflammation, tissue oxygenation, and microvascular perfusion, improving symptoms and the local wound environment without consistently inducing complete osseous regeneration, a notion not consistently verified by radiologic or histologic endpoints in MRONJ cohorts [77,78]. Disease stage at initiation appears critical. Retrospective analyses and reviews indicate that early or limited-stage MRONJ is more likely to benefit, whereas extensive necrosis shows minimal response; continuation of antiresorptives during HBOT—identified in a prospective series—may further diminish efficacy [62]. Stage-dependent nuances and the influence of surgical technique, infection control, and concomitant pharmacotherapy are emphasized in recent evidence syntheses [83,84]. Methodologically, non-uniform diagnostic criteria and outcome definitions (inconsistent staging systems; variable definitions of “healing”) hinder data comparability and complicate evidence-based guideline development, while economic/logistical constraints (cost, access, multi-week schedules) have contributed to underpowered or prematurely terminated studies [78]. Although larger evidence syntheses generally do not demonstrate a consistent benefit of HBOT in MRONJ, smaller uncontrolled series and case reports frequently describe symptomatic and soft-tissue improvements (e.g., pain relief, reduced swelling, improved mucosal coverage, quality of life) within multimodal protocols [62]. Taken together, this supports HBOT’s role in optimizing the local healing milieu rather than driving durable bone regeneration. Narrative and integrative reviews converge on multidisciplinary, case-by-case use while underscoring persistent evidence gaps [78,79,80,84].

In summary, HBOT may accelerate symptom improvement, reduce pain, and support soft tissue healing when used alongside surgery and antibiotics in selected MRONJ patients; however, effects on long-term osseous regeneration are inconsistent, and certainty remains very low to low across systematic reviews. Accordingly, HBOT should not be used routinely but may be considered case by case in early or limited disease, particularly in centers with hyperbaric expertise [78,84]. Future multicenter RCTs with standardized HBOT parameters, harmonized outcome definitions, and long-term follow-up are needed to clarify its true therapeutic value [77,78,79,83]. Building on these findings, the following section focuses on healing outcomes after dental extractions and implant placement in medically compromised or irradiated patients, where HBOT has similarly been evaluated as an adjunctive therapy.

### 5.3. Healing After Implantation and Extractions in Compromised Patients

Patients with compromised wound biology—such as post-extraction sockets, metabolic dysregulation (e.g., diabetes) or previous head-and-neck irradiation—are at increased risk of delayed alveolar healing and impaired implant outcomes. HBOT may enhance early repair by improving tissue oxygenation, angiogenesis, and osteogenesis; however, the extent to which these biological effects translate into clinical benefit remains uncertain. Below, we summarize animal and clinical evidence independently and integrate the implications for practice.

#### 5.3.1. Evidence from In Vivo Studies

Post-extraction healing and ridge preservation. In beagle dogs, HBOT (100% O_2_, 2.4 ATA, 90 min/day, 5×/week for 8 weeks) led to significantly better preservation of alveolar ridge width and buccal contour, higher bone mineral density, and up-regulation of VEGF and BMP-2, indicating intensified angiogenesis, early osteoid formation and mineralization [85]. Early osseointegration under diabetic conditions (rabbit model). In alloxan-diabetic rabbits, HBOT increased bone–implant contact (BIC) at 4 weeks, but no between-group differences persisted at 8 weeks, and RFA-based implant stability did not differ at either time point—suggesting an early-phase, time-limited benefit [86]. In alloxan-diabetic rats, pre- or post-implant HBOT courses increased BIC at 28 days in diabetic animals—approaching values of healthy controls—consistent with partial compensation of the diabetic impairment in early bone–implant integration [87]. Across models, HBOT consistently enhances early-phase events—angiogenesis (↑VEGF), matrix deposition/mineralization (↑BMP-2), and BIC—under hypoxic or metabolically adverse conditions; durability of these gains into long-term mechanical stability remains uncertain [85,87].

#### 5.3.2. Evidence from Clinical Studies

Implant placement in irradiated patients. In a pooled meta-analysis by Chambrone et al. (2022), the relative risk of implant failure with adjunctive HBOT compared with standard care was 1.28 (*p* = 0.80), indicating a non-significant difference and no survival advantage [88]. The Cochrane Review identified one small RCT (26 patients) with no differences between HBOT and no-HBOT in implant/prosthesis failures, postoperative complications or denture satisfaction; overall certainty was very low [89]. A later meta-analysis reaffirmed no survival advantage of HBOT in irradiated patients [90]. Contemporary analyses confirm lower implant survival in irradiated than in non-irradiated jaws and identify jaw location (mandible > maxilla), radiation dose, and surgical timing as primary determinants of outcome. HBOT was not identified as a modifier of implant survival [65]. Brief note on inflammatory–metabolic compromise. While detailed periodontology evidence is presented in Section 5.4, small clinical studies in high-inflammation/metabolic-risk settings suggest short-term adjunctive benefits of HBOT that are biologically consistent with early-phase in vivo effects; these signals do not establish improved implant survival and require larger, longer-term RCTs before translation to routine implant indications.

#### 5.3.3. Synthesis and Clinical Implications

Experimental and preclinical data consistently demonstrate that HBOT enhances early phases of bone repair through improved tissue oxygenation, angiogenesis, and matrix mineralization. In animal models, HBOT accelerates post-extraction healing and ridge preservation in beagle dogs—showing increased bone mineral density and up-regulation of VEGF and BMP-2—and improves early BIC in diabetic models [85,86,87]. These findings are derived from controlled, short-term settings, where the biological environment and HBOT exposure are uniform and the endpoints are biochemical or histomorphometric markers directly responsive to improved oxygen availability. In contrast, clinical studies in irradiated human jaws have yielded neutral results. Meta-analyses involving over 10,000 implants show that HBOT does not improve implant survival in irradiated bone [88]. The only available randomized controlled trial also found no differences between HBOT and control groups in implant or prosthesis failures, postoperative complications, or patient satisfaction [89]. Later reviews confirmed these findings [65,90]. The discrepancy between the in vivo and clinical results can be explained by three main factors. First, animal experiments evaluate short-term biological responses (e.g., VEGF expression, BIC, osteoid formation), while clinical trials measure long-term mechanical survival under multifactorial influences such as tissue perfusion, radiation damage, systemic health, and prosthetic load. Second, human studies are confounded by heterogeneity of HBOT protocols (pressure, duration, number of sessions, pre- vs. postoperative application) and variability in radiation dose, timing, and jaw anatomy. Third, animal models reflect idealized healing conditions, whereas irradiated human bone exhibits irreversible vascular injury, fibrosis, and reduced regenerative potential—biological limitations that HBOT may not fully overcome. Thus, while HBOT clearly stimulates early healing pathways, this does not necessarily translate into sustained osseointegration or implant survival in complex clinical contexts.

#### 5.3.4. Study Limitations and Clinical Constraints

Existing clinical evidence is constrained by a limited number of randomized controlled trials, small sample sizes, and methodological heterogeneity. Most available data are retrospective or observational, and HBOT regimens differ substantially between centers in terms of pressure (2.0–2.5 ATA), number of sessions (10–30), and perioperative timing. Furthermore, studies demonstrating benefit are limited to early biological markers—angiogenic activity, BIC, and short-term radiographic bone gain—without validation in long-term endpoints such as implant survival or functional rehabilitation [88,89,90]. In summary, HBOT exhibits strong experimental evidence for enhancing early osseous healing and angiogenesis under hypoxic or metabolically compromised conditions, supporting its role as a biological adjunct during the early healing phase [85,86,87]. However, current clinical evidence in irradiated patients does not confirm a significant improvement in implant survival, and the magnitude of benefit likely depends on local perfusion, tissue quality, and timing rather than HBOT alone [65,88,90]. Therefore, HBOT should be regarded as promising yet unproven in implantology for compromised patients—its biological plausibility is strong, but translational validation remains incomplete. Large, well-designed, multicenter RCTs with standardized HBOT protocols and long-term follow-up are required to determine its definitive efficacy and cost-effectiveness. Section 5.4, extends this discussion toward HBOT’s potential in periodontal and regenerative surgical contexts, where controlled angiogenic and osteogenic stimulation may complement advanced biomaterials and regenerative therapies.

### 5.4. Regenerative Surgery and Periodontology

Periodontal and regenerative surgical environments are characterized by chronic inflammation, microbial biofilm accumulation, and hypoxia, which together impair angiogenesis and delay tissue repair. HBOT enhances oxygen delivery to hypoperfused tissues, upregulates pro-regenerative mediators such as VEGF and BMP-2, and stimulates fibroblast proliferation and osteoblastic differentiation. HBOT also exhibits bacteriostatic effects against anaerobic periodontal pathogens and modulates inflammatory cytokine profiles, reducing oxidative stress. These mechanisms form a biologically coherent rationale for adjunctive HBOT use in regenerative periodontology and oral surgery [91]. The biological processes described in Section 5.3—angiogenesis, osteoid formation, and mineralization—also provide the mechanistic foundation for potential HBOT benefits in periodontal tissue regeneration.

#### 5.4.1. Evidence from Non-Surgical Periodontal Therapy (SRP ± HBOT)

Clinical data primarily originate from randomized or controlled studies investigating HBOT as an adjunct to scaling and root planing (SRP). In a four-arm randomized clinical trial, Chen et al. evaluated patients with aggressive periodontitis treated with HBOT alone, SRP alone, combined HBOT + SRP, or no therapy. Adjunctive HBOT significantly reduced probing depth (PD), gingival inflammation, and bleeding on probing (BOP) and decreased anaerobic bacterial counts, with the combined HBOT + SRP group achieving the most sustained improvement after two years [92]. Similarly, a prospective randomized study of 71 patients with mild to moderate periodontitis demonstrated that 20 sessions of adjunctive HBOT produced greater improvement in Oral Hygiene Index–Simplified (OHI-S), Sulcus Bleeding Index (SBI), tooth mobility, and PD reduction compared with SRP alone [93]. In a pilot trial including patients with type 2 diabetes and chronic periodontitis, Latusek et al. reported that 30 sessions of HBOT after SRP led to larger PD reduction and greater clinical attachment level (CAL) gains, while both groups showed similar BOP reduction. The authors concluded that HBOT may selectively enhance periodontal attachment repair in metabolically compromised patients [94]. Additional evidence from small prospective studies supports these findings. Nogueira-Filho et al. observed greater PD reduction and lower anaerobic bacterial counts in patients receiving adjunctive HBOT compared with SRP alone [95]. Lombardo et al. reported that 10 HBOT sessions following full-mouth ultrasonic debridement resulted in lower BOP and delayed recolonization of anaerobes compared with mechanical therapy alone [96].

Collectively, these studies indicate that HBOT improves short-term clinical and microbiological outcomes when combined with SRP, although evidence on long-term stability and tooth preservation remains limited.

#### 5.4.2. Evidence from Regenerative and Surgical Applications

Clinical data regarding HBOT in regenerative or implant-related periodontal therapy remain sparse. A case report described the management of advanced periodontitis with implant rehabilitation using advanced platelet-rich fibrin (A-PRF) and perioperative HBOT. After six months, implant stability was satisfactory, and at fourteen months, complete clinical remission of periodontitis was documented. The authors attributed these outcomes to enhanced angiogenesis, improved osseointegration, and antimicrobial effects of HBOT [97]. However, as single-case evidence, these findings should be interpreted cautiously. At a broader level, a systematic review analyzing studies on periodontitis, MRONJ, and ORN found heterogeneous results for HBOT, with methodological limitations precluding meta-analysis. While biological plausibility was confirmed, the authors emphasized the need for standardized treatment parameters and controlled designs [98]. A complementary narrative review reached similar conclusions, highlighting consistent short-term periodontal improvements but noting the lack of uniform HBOT protocols and follow-up beyond one year [99].

#### 5.4.3. Synthesis and Periodontal Implications

Taken together, the available evidence demonstrates a biologically plausible, mechanistically consistent, and clinically promising role of HBOT in enhancing periodontal healing. Across randomized and controlled studies, HBOT consistently produced short-term improvements in PD, CAL, and inflammatory indices, findings that parallel its known biological effects—increased angiogenesis, enhanced osteoblastic differentiation, and suppression of anaerobic microbial load. These convergent preclinical and clinical signals suggest that HBOT may accelerate soft- and hard-tissue repair, particularly under conditions of metabolic or inflammatory compromise. Although current clinical evidence remains heterogeneous, reflecting small sample sizes, single-center designs, and variation in HBOT protocols (2.0–2.5 ATA, 10–30 sessions), the overall direction of results is favorable. Differences in patient selection, disease severity, and timing of therapy may explain some inconsistencies across studies. Importantly, the available data indicate that the biological benefits of HBOT are reproducible, even if their translation into long-term functional outcomes (attachment stability, bone gain, tooth or implant survival) has not yet been demonstrated in large, controlled trials [98].

#### 5.4.4. Methodological Limitations in Periodontal Applications

The clinical evidence base is constrained by a small number of randomized trials, limited sample sizes, and inconsistent reporting of key parameters such as pressure, exposure time, and total session count. Most studies emphasize early clinical or microbiological outcomes without long-term follow-up beyond six to twelve months. Furthermore, none have systematically evaluated patient-reported outcomes or cost-effectiveness. Variability in study populations (e.g., diabetic vs. non-diabetic, aggressive vs. chronic periodontitis) further limits comparability. These methodological issues collectively prevent robust meta-analytic conclusions and weaken external validity. The evidence consistently supports short-term clinical and microbiological benefits of HBOT combined with scaling and root planing, though long-term outcomes and cost-effectiveness remain to be clarified in larger multicenter trials. Table 4 summarizes the key clinical and review studies evaluating HBOT in periodontal and regenerative applications, including the randomized and controlled trials discussed above, as well as higher-level syntheses.

While HBOT alone does not replace conventional debridement or regenerative surgery, it enhances oxygen-dependent cellular activity, promotes angiogenesis, and accelerates early connective tissue repair, leading to measurable short-term gains in probing depth and clinical attachment. Its low invasiveness, favorable safety profile, and potential synergy with biomaterials such as platelet concentrates or bone substitutes make it an attractive option for integration into regenerative protocols. However, before HBOT can be adopted as a standard adjunct in periodontology, multicenter randomized clinical trials with standardized parameters (pressure, duration, timing) are needed to confirm long-term stability and cost-effectiveness.

In summary, HBOT appears to be a promising adjunctive strategy that biologically supports early healing in periodontal therapy, particularly in metabolically compromised patients or in regenerative settings. While it cannot replace conventional mechanical or surgical approaches, it may enhance regenerative potential when combined with biomaterials or biologic modifiers. These encouraging findings justify further multicenter, long-term randomized trials to define optimal HBOT parameters, confirm durability of outcomes, and clarify its role in comprehensive regenerative periodontal therapy. Collectively, the regenerative effects of HBOT observed in periodontal therapy—driven by enhanced angiogenesis and modulation of inflammation—suggest broader applicability in bone disorders marked by hypoxia and deficient vascularity. The following section therefore addresses rare maxillomandibular conditions where these mechanisms may provide therapeutic benefit, including osteopetrosis-associated and pediatric chronic osteomyelitis.

### 5.5. Rare Maxillomandibular Conditions Potentially Benefiting from HBOT

The preceding Section 5.1, Section 5.2, Section 5.3 and Section 5.4 demonstrated that HBOT may enhance tissue repair in various oral contexts—from wound healing and osseointegration to periodontal regeneration—through mechanisms involving improved oxygen delivery, angiogenesis, and modulation of inflammation. Extending this biological rationale, HBOT has also been explored in rare maxillomandibular disorders characterized by severe hypoxia and impaired vascularization, such as osteopetrosis-associated osteomyelitis and primary chronic osteomyelitis. However, whereas the preceding sections summarized moderate- to high-certainty evidence from experimental studies, clinical trials, and meta-analyses, the available data for these rare maxillofacial bone disorders are limited to very low-certainty observations, consisting almost exclusively of isolated case reports and small case series with heterogeneous HBOT protocols and variable outcomes. These conditions therefore represent a biologically plausible yet clinically unconfirmed extension of HBOT application within maxillofacial surgery. Certain rare maxillomandibular entities, particularly osteopetrosis-associated osteomyelitis and pediatric primary chronic osteomyelitis, share pathophysiological features of hypovascular, hypoxic bone with limited reparative potential, rendering them theoretically suitable candidates for adjunctive HBOT; nevertheless, current evidence remains confined to isolated clinical observations without controlled validation [54,100,101,102].

#### 5.5.1. Osteopetrosis-Associated Osteomyelitis

Osteopetrosis is a hereditary disorder of osteoclast function leading to increased bone density and reduced marrow spaces [103]. The autosomal dominant form (ADO, Albers-Schönberg disease) typically manifests in adolescence or adulthood, with mandibular osteomyelitis developing in approximately 10% of patients, often after dental extraction or secondary infection due to poor bone vascularity [101]. Reported outcomes of HBOT in this context are heterogeneous. In a 26-year-old male with ADO, 30 HBOT sessions (2.4 ATA, 90 min) combined with antibiotics and debridement failed to achieve complete remission [101]. In contrast, a more recent case reported sustained remission of refractory maxillary osteomyelitis associated with osteopetrosis after combined surgical and HBOT management, with no recurrence over two years [104]. Another case of osteoclast-poor osteopetrosis with RANKL mutation demonstrated repeated relapses despite HBOT, although hyperoxia was suggested to enhance antibiotic efficacy and partially suppress infection [105]. Overall, the therapeutic response to HBOT in osteopetrosis-associated osteomyelitis remains inconsistent, likely influenced by disease subtype, vascularity, and concomitant surgical management. No standardized HBOT protocol has been established for this indication.

#### 5.5.2. Primary Chronic Osteomyelitis of the Jaws

Primary chronic osteomyelitis (PCO) is a non-suppurative inflammatory disorder affecting the mandible, predominantly in children and adolescents. In a small series of four pediatric patients, adjunctive HBOT following surgical debridement and antibiotic therapy resulted in three patients remaining symptom-free for 20–74 months [102]. However, due to the absence of controls and the small sample size, these results are exploratory. Broader reviews emphasize that HBOT should be considered an adjunct to anti-inflammatory medication and surgical decortication, not a primary treatment modality [100].

#### 5.5.3. Other Rare Maxillofacial Entities

HBOT has also been sporadically applied in other maxillomandibular conditions characterized by impaired vascularization. Successful management of zygomatic bone osteomyelitis with 30 HBOT sessions (2.5 ATA, 90 min) combined with conservative sequestrectomy has been reported [106]. In infected cemento-osseous dysplasia (COD), standard management involves antibiotics and debridement; only isolated reports mention adjunctive HBOT with uncertain benefit [107,108]. Rare cases of chronic mandibular osteomyelitis due to *Granulicatella adiacens* have been successfully treated with surgery and antibiotics, though HBOT use remains anecdotal [109].

#### 5.5.4. Synthesis and Clinical Implications

Across these rare conditions, HBOT is consistently used as an adjunctive rather than primary therapy, aiming to enhance tissue oxygenation, stimulate angiogenesis, and improve antibiotic delivery within hypoxic bone. Case-level evidence suggests possible symptomatic improvement or infection control in selected patients, particularly when combined with meticulous surgical and antimicrobial management. However, no controlled trials have validated its efficacy, and no standardized HBOT parameters (pressure, duration, or timing) have been defined.

#### 5.5.5. Evidence Limitations in Rare Maxillomandibular Conditions

The current evidence base regarding HBOT use in rare maxillomandibular disorders is extremely limited, consisting solely of case reports and small case series. No randomized or prospective trials have been conducted, and most publications lack standardized HBOT parameters (pressure, duration, and number of sessions). Furthermore, heterogeneity in surgical management, antibiotic regimens, and diagnostic criteria impedes meaningful comparison across studies. Publication bias is likely, as successful outcomes are more frequently reported than failures. Consequently, the true efficacy, safety, and cost-effectiveness of HBOT in these indications remain uncertain.

In summary, HBOT represents a biologically plausible yet clinically unverified adjunct in the management of rare maxillomandibular conditions such as osteopetrosis-associated osteomyelitis and primary chronic osteomyelitis. While isolated cases report favorable responses—particularly when HBOT is combined with surgery and antibiotics—these findings are based on very low-certainty evidence. To establish its role, multicenter, prospective studies with standardized HBOT protocols, long-term follow-up, and clearly defined clinical endpoints are required. Collectively, the findings from Section 5.1, Section 5.2, Section 5.3, Section 5.4 and Section 5.5 demonstrate that HBOT exerts measurable biological effects across a spectrum of oral and maxillofacial conditions; however, the strength of evidence progressively declines from experimental to rare clinical applications. Section 7 integrates these findings to critically evaluate HBOT’s translational relevance, therapeutic boundaries, and future research priorities in dental and maxillofacial medicine.

## 6. Contraindications for Patient Eligibility for HBOT

Patient qualification for HBOT requires a careful analysis of contraindications, which may influence both the safety and efficacy of therapy. In the clinical literature, untreated pneumothorax is considered the main absolute contraindication to HBOT, since exposure to increased pressure may transform it into a tension pneumothorax, posing an immediate life-threatening risk [110,111]. Although many sources state that untreated pneumothorax is the only absolute contraindication to HBOT, clinical practice and recent expert reports indicate that the presence of intraocular gas is often treated as a de facto absolute contraindication, despite being classified as relative. Gases such as perfluoropropane (C_3_F_8_), sulfur hexafluoride (SF_6_), and perfluoroethane (C_2_F_6_), used as long-acting tamponades in vitreoretinal surgery, expand significantly under hyperbaric conditions. During decompression, this expansion can cause a dramatic increase in intraocular pressure (IOP), resulting in acute ischemia of the optic nerve, retina, and anterior ocular structures, potentially leading to irreversible blindness even after a single HBOT session [110]. For this reason, the presence of intraocular gas not fully absorbed on imaging is considered an “absolute-conditional” contraindication—one that may only be overridden in life-threatening situations where withholding HBOT poses a greater risk (e.g., severe carbon monoxide poisoning, necrotizing fasciitis) [112]. Relative contraindications include selected chemotherapeutic agents and comorbidities that may increase the risk of severe complications:

Bleomycin: associated with pulmonary fibrosis and heightened pulmonary risk during HBOT. Case series (Torp 2012, n = 15) reported no acute pulmonary complications under a cautious protocol (chest X-ray, spirometry, blood gases, test session at 2.0 ATA), even within 6 months of administration [113]. Reviews recommend delaying HBOT for ≥3–4 months post-bleomycin, with careful pulmonary assessment [111,113]. Current evidence considers this a relative, controversial contraindication [111].

Doxorubicin: cardiotoxicity is the primary concern. While animal data are inconclusive, practical guidelines recommend waiting at least 72 h post-infusion before HBOT initiation [111,114]. Earlier reports (Karagöz et al., 2008) categorized it as absolute, but newer studies suggest a possible cardioprotective effect of HBOT [115].

Cisplatin: impairs fibroblast and collagen activity, potentially reducing wound healing and HBOT efficacy. It is now considered a relative contraindication, with cautious use permitted in urgent cases [111].

Among comorbidities, respiratory diseases are especially relevant: COPD, asthma, pulmonary bullae, and emphysema increase the risk of barotrauma and pneumothorax. Prospective data indicate no significant deterioration in lung function after 20–60 HBOT sessions in appropriately selected patients [116,117]. Thus, COPD and asthma are relative contraindications, requiring pulmonary imaging and spirometry prior to HBOT. Severe emphysema with bullae carries particularly high risk [110,118]. Controlled asthma is not necessarily contraindicated, but careful evaluation is advised. Diabetes mellitus (uncontrolled): HBOT should be deferred if pre-treatment capillary glucose is ≥300 mg/dL (≈16.7 mmol/L) or <100 mg/dL (≈5.6 mmol/L)**;** glycemia must be stabilized prior to chamber exposure to minimize the risk of ketoacidosis or hypoglycemia [32].

ENT-related conditions, such as chronic sinusitis, upper respiratory infections, or Eustachian tube dysfunction (ETD), can cause middle ear and sinus barotrauma. The incidence of middle ear barotrauma (MEB) ranges widely, being highest in patients unable to perform equalization maneuvers. Altered mental status has been identified as an independent risk factor for MEB in carbon monoxide poisoning patients undergoing HBOT [119]. Careful otolaryngologic assessment, otoscopy, pressure equalization testing (Valsalva, Toynbee), and in some cases ENT interventions (e.g., tympanostomy tubes) are recommended [114,120]. Claustrophobia poses a functional contraindication, particularly in monoplace chambers. Strategies include use of multiplace chambers, anxiolytic techniques, or pharmacologic support. In refractory cases where anxiety prevents chamber entry, HBOT may be unfeasible [110,118]. Epilepsy or seizure history may represent a relative contraindication, as hyperoxia can induce oxygen toxicity seizures. Retrospective data show a very low incidence (1 seizure in >600 HBOT sessions in epileptic patients) with appropriate monitoring [121]. Mechanisms include increased CNS oxygen levels, ROS production, and reduced seizure threshold [114]. Decompensated heart failure with LVEF < 35% poses a risk due to increased vascular resistance and hemodynamic load under HBOT, potentially precipitating acute pulmonary edema. While data are limited, this is considered a relative contraindication requiring cardiology evaluation [122]. Table 5 presents an overview of the major contraindications to HBOT, classified by their nature and supported by the cited literature.

## 7. Discussion

HBOT represents a biologically sound adjunctive strategy in oral and maxillofacial surgery, particularly in procedures where wound healing is compromised by hypoxia, infection, or vascular insufficiency. By substantially increasing tissue oxygen tension, HBOT enhances oxygen diffusion into poorly perfused bone and soft tissue, stimulates angiogenesis, improves fibroblast and collagen activity, and modulates inflammatory and immune responses. These mechanisms are directly relevant to the oral environment, which is frequently challenged by ischemic bone following radiotherapy, chronic inflammation in MRONJ, or impaired microcirculation in systemic conditions such as diabetes. From a clinical perspective, the present synthesis shows that HBOT consistently improves early wound-healing parameters, reduces pain and soft-tissue inflammation, and accelerates epithelialization. However, robust long-term outcomes—such as complete bone regeneration, durable mucosal coverage, or implant survival—remain inconsistent. In ORN, the most rigorously designed randomized trials, including the HOPON study, did not confirm a prophylactic advantage for HBOT prior to dentoalveolar surgery. Therapeutically, however, observational data and small prospective series suggest that HBOT may be most effective in early or intermediate stages of ORN, where hypoxia and inflammation dominate but irreversible necrosis has not yet developed. In MRONJ, the biological rationale is equally strong. A controlled clinical trial and multiple case series demonstrate faster clinical improvement, pain reduction, and improved tissue quality when HBOT accompanies standard surgery and antibiotic therapy. Nevertheless, rates of complete mucosal closure and long-term recurrence prevention remain inconsistent. For implant-related surgery, translational studies demonstrate that HBOT enhances angiogenic and osteogenic signaling—through increased VEGF and BMP-2 expression—but these findings have not yet translated into improved implant survival in irradiated bone. In periodontology, randomized controlled trials by Chen et al. and Latusek et al. confirm short-term benefits of HBOT combined with scaling and root planing, particularly in patients with diabetes or aggressive periodontitis, yet the persistence of these effects beyond 6–12 months remains uncertain [92,94].

The limitations of current evidence are primarily methodological. Published studies employ highly heterogeneous HBOT protocols (2.0–2.8 ATA, 10–60 sessions, variable duration and timing), and preventive and therapeutic indications are frequently combined. Many trials are underpowered, with small cohorts and short follow-up. Confounding from concomitant therapies (antibiotics, PENTO/PENTOCLO, platelet concentrates, or laser therapy) is rarely controlled. Moreover, definitions of “healing” differ substantially—ranging from mucosal closure to absence of infection or radiological resolution—limiting comparability across studies. Few trials have incorporated patient-reported outcomes or cost-effectiveness analyses, which are essential for clinical decision-making. Despite these shortcomings, HBOT offers distinct clinical advantages when used judiciously. For the practicing dentist or oral surgeon, its greatest potential lies in carefully selected clinical scenarios. In early-stage osteoradionecrosis following head and neck radiotherapy, HBOT can serve as an adjunct to conservative surgical debridement and infection control, helping to improve mucosal closure and reduce pain. In MRONJ, particularly in stages I–II with soft-tissue inflammation or localized necrosis, HBOT may accelerate symptom resolution and enhance tissue repair when combined with antibiotic therapy. In complex extractions, reconstructive procedures, or implant placement in irradiated or ischemic regions, HBOT may promote healing by improving local oxygen tension and perfusion. Similarly, in patients with metabolic compromise—such as diabetes or peripheral vascular disease—it can transiently enhance microcirculation and bacterial control, supporting periodontal regeneration. Finally, in refractory osteomyelitis, improved tissue oxygenation augments the activity of oxygen-dependent antibiotics and host immune responses, potentially shortening recovery and reducing recurrence risk.

Conversely, HBOT should not be applied routinely as prophylaxis against ORN, nor expected to improve implant survival in irradiated bone. It cannot replace surgical debridement, infection control, or standard pharmacologic management. Its use should be restricted to well-selected cases in which the biological deficit—tissue hypoxia—can realistically be reversed. Patient eligibility for HBOT must be carefully assessed, as contraindications can substantially affect both the safety and efficacy of treatment. While untreated pneumothorax remains the only absolute contraindication, several conditions—such as intraocular gas tamponade, severe emphysema with bullae, uncontrolled diabetes, or recent exposure to bleomycin—require individualized risk–benefit evaluation prior to therapy initiation. For future research, methodological refinement is crucial. Multicenter randomized trials with standardized HBOT parameters, clear distinction between prophylactic and therapeutic indications, and unified outcome measures are required. Studies should integrate objective imaging (perfusion MRI, CBCT perfusion mapping), biomarkers of oxygen response (VEGF, HIF-1α), and patient-centered endpoints such as pain, function, and oral health–related quality of life. Long-term follow-up and cost analyses are needed to define when HBOT is clinically and economically justified.

In summary, HBOT demonstrates convincing biological plausibility and short-term clinical benefits across several domains of dental and maxillofacial surgery. When applied selectively—particularly in early osteoradionecrosis, MRONJ, refractory osteomyelitis, and compromised wound healing—it can significantly support recovery and improve patient comfort. However, routine prophylactic use and reliance on HBOT as a stand-alone therapy are not supported by current high-quality evidence. The future of HBOT in dentistry will depend on rigorous, standardized trials that integrate mechanistic, clinical, and patient-reported outcomes to delineate its true therapeutic value.

## 8. Conclusions

1. HBOT may be a biologically justified adjunctive therapy in dental and maxillofacial surgery, acting through enhanced tissue oxygenation, stimulation of angiogenesis, modulation of inflammation, and improved antimicrobial defense.
2. Clinical benefits may be most evident in early and high-risk conditions, including ORN, MRONJ, and refractory osteomyelitis, where HBOT can accelerate granulation, reduce pain, and support mucosal closure.
3. Evidence for long-term bone regeneration, prevention of complications, and implant survival remains limited. Routine prophylactic use of HBOT in irradiated or high-risk patients is not currently supported by high-quality data.
4. HBOT should be applied selectively, as an adjunct to established surgical and pharmacologic protocols, particularly when wound healing is compromised by local ischemia, infection, or systemic comorbidities such as diabetes.
5. Clinical success depends on individualized patient selection, standardized protocols, and interdisciplinary coordination between oral surgeons, radiation oncologists, and hyperbaric specialists.
6. Patient selection should always include screening for contraindications such as pneumothorax, intraocular gas, or severe pulmonary disease, as these factors may preclude safe HBOT administration.
7. Future research should focus on high-quality multicenter randomized trials with uniform HBOT parameters, longer follow-up, and inclusion of patient-reported and cost-effectiveness outcomes.
8. In rare maxillomandibular conditions such as osteopetrosis-related or primary chronic osteomyelitis, current evidence is anecdotal; HBOT should be regarded as experimental and considered only on a case-by-case basis.
9. Overall, HBOT may represent a physiologically coherent and clinically valuable support modality for complex wound healing in dentistry, effective in selected indications but not yet established as a universal standard of care.

## Figures and Tables

**Table 1:** Summary of the key mechanisms of action of HBOT.

Mechanism	Description	Key Effects	References
Increased oxygen solubility (Henry’s law)	Elevated pressure (2–3 ATA) leads to a tenfold increase in plasma oxygen content, enabling diffusion into hypoxic tissues.	Improved perfusion in ischemic areas; plasma oxygen concentration may reach approximately 4–6 mL/dL at ~2.0 ATA, depending on temperature and hemoglobin concentration.	Ortega et al., 2021 [23]; Heyboer et al., 2017 [6]; Jeyaraman et al., 2023 [26]
Induction of oxidative stress (ROS/RNS)	Generation of reactive oxygen/nitrogen species as molecular signaling mediators, modulation of NF-κB/IκBα pathway.	Reduction in pro-inflammatory cytokines (observed in studies using ~2.0–2.5 ATA for 60–90 min, single or limited sessions); this effect may invert with higher pressures or prolonged exposure	De Wolde et al., 2021 [24], Fosen & Thom, 2014 [44]
Activation of HIF-1α	HBOT, through alternating hyperoxia and normoxia, secondarily stabilizes HIF-1α and increases VEGF and SDF-1 expression.	Stimulation of angiogenesis and fibroblast proliferation.	Lindenmann et al., 2022 [42]; Sunkari et al., 2015 [27]
Effects on fibroblasts and collagenogenesis	Enhanced fibroblast proliferation, increased type III collagen synthesis, and extracellular matrix reorganization.	Accelerated wound healing, improved granulation tissue architecture.	Kang et al., 2004 [39]; Huang et al., 2020 [40]; Růžička et al., 2021 [47]
Immunomodulatory and antibacterial effect	Increased ROS generation in neutrophils, suppression of NF-κB.	Enhanced phagocytosis, reduced infection, transition toward proliferative phase.	Lerche et al., 2017 [28]; Capó et al., 2023 [29]; Gill & Bell, 2004 [7]

**Table 2:** Summary of key clinical studies, systematic reviews, and guidelines evaluating hyperbaric oxygen therapy for the prevention and management of ORN.

Study	Study Type/Population	Intervention	Key Outcomes	Conclusions and Limitations
Lin et al., 2023 (Cochrane) [67]	Systematic review of late radiation tissue injury/(soft tissue + osseous)	HBOT vs. no HBOT	Benefit for selected soft-tissue LRTI (e.g., cystitis, proctitis); uncertain effect for ORN	Moderate–low certainty; heterogeneous RCTs; inconsistent endpoints
El-Rabbany et al., 2019 (Cochrane) [68]	Systematic review of ORN prevention/(4 RCTs; ~n = 340)	Peri-operative HBOT vs. standard dental care	No clear prophylactic advantage of HBOT	Very low certainty; small trials; underpowered
Shaw et al., 2019 (HOPON RCT) [20]	Multicenter RCT/n = 144; dentoalveolar surgery in irradiated mandible	HBOT 2.4 ATA × 30 sessions (pre + post) vs. standard care	ORN: 6.4% (HBOT) vs. 5.7% (control); NS	No preventive benefit; low baseline ORN incidence in both arms
Jenwitheesuk et al., 2018 [69]	Retrospective clinical study/n = 84; ORN stages I–III	HBOT as adjunct (2.4 ATA × 30)	Improved healing in stages I–II; limited effect in stage III	Non-randomized; single-center; variable co-interventions
Mohandas et al., 2023 (Thieme SR) [70]	Systematic review/6 studies; 3 RCTs; ~n = 300	HBOT ± surgery/antibiotics vs. control	Possible benefit in early ORN or as adjuvant to surgery	Low–moderate quality; inconsistent healing definitions
Geçkil et al., 2025 [71]	Meta-analysis/treatment strategies for mandibular ORN	HBOT + surgery vs. single-modality therapy	Combination superior to HBOT or surgery alone	Heterogeneous protocols/outcomes; limited high-quality data
Peterson et al., 2024 (ISOO–MASCC–ASCO Guideline) [74]	Evidence-based clinical guideline/-	Review of SRs/MAs + expert consensus	HBOT not recommended routinely; consider case-by-case (early ORN/adjunct)	Few modern RCTs; emphasis on prevention and optimized dental/surgical care
Fritz et al., 2025 (Review) [72]	Narrative state-of-the-art review/mandibular ORN	—	Paradigm shift away from routine HBOT; emphasis on PENTO/PENTOCLO and reconstructive surgery	Narrative synthesis; integrates recent SRs and guidelines
Dang et al., 2023 (Aust Dent J) [73]	Retrospective audit + narrative review/n = 121 extractions post-RT	Prophylactic HBOT before/after extractions	ORN ≈ 9%; incomplete HBOT adherence; no clear efficacy signal	Heterogeneous cohort; confounding by indication

Abbreviations: LRTI—Late Radiation Tissue Injury; ISOO—International Society of Oral Oncology; MASCC—Multinational Association of Supportive Care in Cancer; ASCO—American Society of Clinical Oncology; PENTO/PENTOCLO—Pentoxifylline + Tocopherol (±Clodronate) therapy; NS—Not Significant; RT—Radiotherapy; QoL—Quality of Life; SDC—Standard Dental Care; EF—Ejection Fraction.

**Table 3:** Summary of key clinical studies and systematic reviews evaluating hyperbaric oxygen therapy as an adjunctive treatment for MRONJ.

Study	Study Type/Population	Intervention	Primary Outcomes	Conclusions and Limitations
Freiberger et al., 2012 [77]	RCT/49 randomized, 46 analyzed; adults with BP-ONJ	HBOT 2.0 ATA, twice daily, 40 sessions + surgery/antibiotics vs. standard care	Clinical improvement: 68% HBOT vs. 38.1% control (*p* = 0.043). Time to first improvement: 39.7 vs. 67.9 weeks (*p* = 0.03). Faster pain resolution (*p* < 0.01). Complete gingival healing: 52% vs. 33.3% (NS).	Significant symptomatic benefit and faster recovery; no significant difference in mucosal closure; single-center, small RCT.
Freiberger et al., 2007 [62]	Prospective case series/n = 16 adults with BP-ONJ	Adjunctive HBOT + standard care (protocol per study)	Clinical improvement: 87.5%; stable remission: 62.5%; recurrence earlier if bisphosphonate use continued (8.5 vs. 20.1 months, *p* = 0.006).	Suggests supportive effect of HBOT in early stages; uncontrolled, small sample; recurrence risk when bisphosphonates continued.
Beth-Tasdogan et al., 2022 (Cochrane) [78]	Systematic review/management of MRONJ (includes Freiberger RCT)	Various interventions (including HBOT as adjunct) vs. standard	Very low–low certainty for most interventions; HBOT evidence limited to one small RCT; no conclusive benefit.	Highest-quality SR available; major heterogeneity and small sample sizes; need for robust modern RCTs.
Govaerts et al., 2020 [79]	Systematic review/adjuvant therapies for MRONJ (laser, L-PRF, fluorescence-guided surgery, HBOT)	Adjuvant + standard care vs. standard	Strongest evidence for laser and L-PRF; HBOT reported in few small heterogeneous case series; overall low evidence quality.	Broad review; risk of bias and heterogeneity; calls for controlled studies comparing adjuvants including HBOT.
Ceponis et al., 2017 [80]	Narrative review/HBOT in osteonecrosis (radiation- and medication-related)	—	Discusses mechanistic rationale (angiogenesis, anti-inflammatory effects); acknowledges clinical and economic controversies; recommends multimodal therapy (surgery + antibiotics + HBOT).	Conceptual synthesis; not a meta-analysis; integrates mechanistic and clinical insights.
Biancardi et al., 2021 [81]	Integrative review/HBOT in MRONJ	—	Signals of improved pain and quality of life in case series; no modern RCTs; evidence uncertain.	Integrative review mainly of case reports; calls for higher-quality studies.
Frutuoso et al., 2024 [83]	Systematic review/MRONJ (case reports and series including HBOT)	Various interventions (case-based)	HBOT may aid healing and symptom relief in selected patients; very low-level evidence (case-based).	SR based on case studies; describes emerging trends but lacks comparative data.
Byrne et al., 2024 [84]	Narrative/clinical review/MRONJ management in cancer patients on BMAs	—	HBOT discussed as optional adjunct; main focus on prevention, surgery, and pharmacologic therapy (e.g., PENTO protocol).	Practical, experience-based guidance; emphasizes prevention and combined care.

Abbreviations: RCT—Randomized Controlled Trial; SR—Systematic Review; L-PRF—Leukocyte- and Platelet-Rich Fibrin; PENTO/PENTOCLO—Pentoxifylline + Tocopherol (±Clodronate) therapy; BMA—Bone-Modifying Agent; NS—Not Significant; QoL—Quality of Life.

**Table 4:** Summary of clinical and review evidence on hyperbaric oxygen therapy in periodontal and regenerative applications.

Study/Year	Study Type/Population	Intervention (HBOT Protocol)	Main Outcomes	Conclusions
Chen T-L et al., 2012 [92]	RCT/60 patients with aggressive periodontitis, 4-arm RCT (HBOT, SRP, HBOT + SRP, control)	100% O_2_, 2.4 ATA, 90 min × 20 sessions	↓ PD, ↓ BOP, ↓ anaerobic flora; greatest effect in HBOT + SRP group	HBOT + SRP significantly improved clinical and microbiological parameters; benefits sustained for >2 years
Burcea A et al., 2022 [93]	RCT/71 patients with mild–moderate periodontitis, two-arm randomization	SRP ± HBOT (20 sessions, 2.4 ATA, 90 min)	Improved OHI-S, SBI, PD reduction, and tooth mobility vs. SRP alone (*p* < 0.05)	Adjunctive HBOT enhanced clinical outcomes after SRP
Latusek K et al., 2023 [94]	Pilot Clinical Trial/14 patients with type 2 diabetes and chronic periodontitis	SRP ± HBOT (30 sessions, 2.5 ATA, 90 min)	↓ PD and ↑ CAL in HBOT group; BOP similar in both groups	HBOT selectively improves attachment gain in metabolically compromised patients
Nogueira-Filho GR et al., 2010 [95]	Controlled Prospective Study/Patients with severe periodontitis (n ≈ 20)	SRP ± HBOT (2.4 ATA, 10 sessions)	↓ PD and ↓ anaerobic bacteria count	HBOT provided short-term clinical and microbiological benefit
Lombardo G et al., 2020 [96]	Pilot Study/Chronic periodontitis patients (n = 20)	Full-mouth ultrasonic debridement ± 10 HBOT sessions	↓ BOP and delayed recolonization of anaerobic flora	HBOT reduced inflammation and microbial recolonization
Giacon TA et al., 2021 [97]	Case Report/Advanced periodontitis with implants treated using A-PRF + HBOT	2.4 ATA, multiple perioperative sessions	Stable implants after 6 months, complete remission after 14 months	Suggests potential synergy of HBOT with A-PRF in bone regeneration
Heck T et al. 2024 [98]	Systematic Review/16 studies (5 RCTs, 11 prospective); periodontitis, MRONJ, ORN	Various HBOT protocols (2.0–2.5 ATA, 10–30 sessions)	Mixed results: methodological heterogeneity precluded meta-analysis	HBOT has strong biological rationale but inconsistent clinical evidence
Winsness A et al. 2024 [99]	Narrative synthesis/HBOT in dentistry and periodontology	Review of clinical and mechanistic evidence	Consistent short-term PD improvements reported; heterogeneous protocols	HBOT is promising but requires protocol standardization and long-term trials

**Table 5:** Contraindications to Hyperbaric Oxygen Therapy.

Clinical Condition or Factor	Type of Contraindication	Clinical Rationale	References
Untreated pneumothorax	Absolute	Risk of tension pneumothorax under increased pressure	Gawdi et al., 2025 [110]; Baude et al., 2024 [111]
Intraocular gas (C_3_F_8_, SF_6_, C_2_F_6_)	Absolute (conditionally permissible)	Rapid rise in intraocular pressure, risk of blindness	Gawdi et al., 2025 [110]
Bleomycin	Relative	Risk of pulmonary toxicity; therapy possible ≥3–4 months after administration	Baude et al., 2024 [111]; Torp et al., 2012 [113]
Doxorubicin	Relative	Potential cardiotoxicity; recommended interval ≥ 72 h	Baude et al., 2024 [111]; Karagöz et al., 2008 [115]
Cisplatin	Relative	Impaired wound healing and fibroblast activity	Baude et al., 2024 [111]
Chronic obstructive pulmonary disease (COPD), pulmonary bullae, emphysema	Relative	Risk of barotrauma, pneumothorax; particularly in emphysema with hyperinflation	Brenna et al., 2022 [116]; Gawdi et al., 2025 [110]
Stable bronchial asthma	Relative	Requires airway reactivity assessment; possible therapy under pharmacological control	DuBose et al., 2023 [118]
Middle ear and sinus pressure disorders (ETD, URTI, sinusitis)	Relative	Risk of middle ear and sinus barotrauma; most common adverse events	Sadri et al., 2022 [114]; Lee et al., 2025 [119]; Lima et al., 2012 [120]
Claustrophobia	Functional	Intolerance of monoplace chamber may prevent therapy	DuBose et al., 2023 [118]; Gawdi et al., 2025 [110]
Epilepsy/history of seizures	Relative	Risk of oxygen-induced seizures, especially in hyperoxia; low but present	Park et al., 2025 [121]; Sadri et al., 2022 [114]
Heart failure (EF < 35%)	Relative	Possible hemodynamic overload and pulmonary edema	Schiavo et al., 2024 [122]

## Data Availability

No new data were created or analyzed in this study. Data sharing is not applicable to this article.

## References

[1] Wilkinson H.N., Hardman M.J. (2020). Wound healing: Cellular mechanisms and pathological outcomes. Open Biol..

[2] Eming S.A., Martin P., Tomic-Canic M. (2014). Wound repair and regeneration: Mechanisms, signaling, and translation. Sci. Transl. Med..

[3] Reinke J.M., Sorg H. (2012). Wound repair and regeneration. Eur. Surg. Res..

[4] Rodrigues M., Kosaric N., Bonham C.A., Gurtner G.C. (2019). Wound Healing: A Cellular Perspective. Physiol. Rev..

[5] Velnar T., Bailey T., Smrkolj V. (2009). The wound healing process: An overview of the cellular and molecular mechanisms. J. Int. Med. Res..

[6] Heyboer M., Sharma D., Santiago W., McCulloch N. (2017). Hyperbaric Oxygen Therapy: Side Effects Defined and Quantified. Adv. Wound Care.

[7] Gill A.L., Bell C.N. (2004). Hyperbaric oxygen: Its uses, mechanisms of action and outcomes. QJM.

[8] Delanian S., Lefaix J.L. (2004). The radiation-induced fibroatrophic process: Therapeutic perspective via the antioxidant pathway. Radiother. Oncol..

[9] Martin M., Lefaix J., Delanian S. (2000). TGF-beta1 and radiation fibrosis: A master switch and a specific therapeutic target?. Int. J. Radiat. Oncol. Biol. Phys..

[10] Barker H.E., Paget J.T., Khan A.A., Harrington K.J. (2015). The tumour microenvironment after radiotherapy: Mechanisms of resistance and recurrence. Nat. Rev. Cancer.

[11] Yarnold J., Brotons M.C. (2010). Pathogenetic mechanisms in radiation fibrosis. Radiother. Oncol..

[12] Tolentino Ede S., Centurion B.S., Ferreira L.H., Souza A.P., Damante J.H., Rubira-Bullen I.R. (2011). Oral adverse effects of head and neck radiotherapy: Literature review and suggestion of a clinical oral care guideline for irradiated patients. J. Appl. Oral Sci..

[13] Faisal M., Berend P.D., Seemann R., Janik S., Grasl S., Ritzengruber A., Mendel H., Jamshed A., Hussain R., Erovic B.M. (2021). Impact of Previous Irradiation on Wound Healing after Negative Pressure Wound Therapy in Head and Neck Cancer Patients-A Systematic Review. Cancers.

[14] Strojan P., Hutcheson K.A., Eisbruch A., Beitler J.J., Langendijk J.A., Lee A.W.M., Corry J., Mendenhall W.M., Smee R., Rinaldo A. (2017). Treatment of late sequelae after radiotherapy for head and neck cancer. Cancer Treat. Rev..

[15] Haubner F., Ohmann E., Pohl F., Strutz J., Gassner H.G. (2012). Wound healing after radiation therapy: Review of the literature. Radiat. Oncol..

[16] Jacobson L., Johnson M., Dedhia D., Solmaz N.-B., Wong A. (2017). Impaired Wound Healing After Radiation Therapy: A Systematic Review of Pathogenesis and Treatment. JPRAS Open.

[17] Singh A., Huryn J.M., Kronstadt K.L., Yom S.K., Randazzo J.R., Estilo C.L. (2022). Osteoradionecrosis of the jaw: A mini review. Front. Oral Health.

[18] Chrcanovic B.R., Reher P., Sousa A.A., Harris M. (2010). Osteoradionecrosis of the jaws--a current overview--part 1: Physiopathology and risk and predisposing factors. Oral Maxillofac. Surg..

[19] Bennett M.H., Feldmeier J., Hampson N.B., Smee R., Milross C. (2016). Hyperbaric oxygen therapy for late radiation tissue injury. Cochrane Database Syst. Rev..

[20] Shaw R.J., Butterworth C.J., Silcocks P., Tesfaye B.T., Bickerstaff M., Jackson R., Kanatas A., Nixon P., McCaul J., Praveen P. (2019). HOPON (Hyperbaric Oxygen for the Prevention of Osteoradionecrosis): A Randomized Controlled Trial of Hyperbaric Oxygen to Prevent Osteoradionecrosis of the Irradiated Mandible After Dentoalveolar Surgery. Int. J. Radiat. Oncol. Biol. Phys..

[21] Annane D., Depondt J., Aubert P., Villart M., Géhanno P., Gajdos P., Chevret S. (2004). Hyperbaric oxygen therapy for radionecrosis of the jaw: A randomized, placebo-controlled, double-blind trial from the ORN96 study group. J. Clin. Oncol..

[22] Ajayi O.D., Gaskill Z., Kelly M., Logue C.J., Hendricksen S.M. (2020). A comparison of two hyperbaric oxygen regimens: 2.0 ATA for 120 minutes to 2.4 ATA for 90 minutes in treating radiation-induced cystitis Are these regimens equivalent?. Undersea Hyperb. Med..

[23] Ortega M.A., Fraile-Martinez O., García-Montero C., Callejón-Peláez E., Sáez M.A., Álvarez-Mon M.A., García-Honduvilla N., Monserrat J., Álvarez-Mon M., Bujan J. (2021). A General Overview on the Hyperbaric Oxygen Therapy: Applications, Mechanisms and Translational Opportunities. Medicina.

[24] De Wolde S.D., Hulskes R.H., Weenink R.P., Hollmann M.W., Van Hulst R.A. (2021). The Effects of Hyperbaric Oxygenation on Oxidative Stress, Inflammation and Angiogenesis. Biomolecules.

[25] Weaver L.K., Hopkins R.O., Chan K.J., Churchill S., Elliott C.G., Clemmer T.P., Orme J.F., Thomas F.O., Morris A.H. (2002). Hyperbaric oxygen for acute carbon monoxide poisoning. N. Engl. J. Med..

[26] Jeyaraman M., Sami A., Nallakumarasamy A., Jeyaraman N., Jain V.K. (2023). Hyperbaric Oxygen Therapy in Orthopaedics: An Adjunct Therapy with an Emerging Role. Indian J. Orthop..

[27] Sunkari V.G., Lind F., Botusan I.R., Kashif A., Liu Z.J., Ylä-Herttuala S., Brismar K., Velazquez O., Catrina S.B. (2015). Hyperbaric oxygen therapy activates hypoxia-inducible factor 1 (HIF-1), which contributes to improved wound healing in diabetic mice. Wound Repair. Regen..

[28] Lerche C.J., Christophersen L.J., Kolpen M., Nielsen P.R., Trøstrup H., Thomsen K., Hyldegaard O., Bundgaard H., Jensen P., Høiby N. (2017). Hyperbaric oxygen therapy augments tobramycin efficacy in experimental *Staphylococcus aureus* endocarditis. Int. J. Antimicrob. Agents.

[29] Capó X., Monserrat-Mesquida M., Quetglas-Llabrés M., Batle J.M., Tur J.A., Pons A., Sureda A., Tejada S. (2023). Hyperbaric Oxygen Therapy Reduces Oxidative Stress and Inflammation, and Increases Growth Factors Favouring the Healing Process of Diabetic Wounds. Int. J. Mol. Sci..

[30] Bosco G., Brizzolari A., Paganini M., Camporesi E., Vezzoli A., Mrakic-Sposta S. (2025). Oxy-inflammation in hyperbaric oxygen therapy applications. Eur. J. Transl. Myol..

[31] Sen S., Sen S. (2021). Therapeutic effects of hyperbaric oxygen: Integrated review. Med. Gas. Res..

[32] Jones M.W., Brett K., Han N., Cooper J.S., Wyatt H.A. (2025). Hyperbaric Physics. StatPearls.

[33] Zhang Y., Zhou Y., Jia Y., Wang T., Meng D. (2023). Adverse effects of hyperbaric oxygen therapy: A systematic review and meta-analysis. Front. Med..

[34] Weaver J., Liu K.J. (2015). Does normobaric hyperoxia increase oxidative stress in acute ischemic stroke? A critical review of the literature. Med. Gas. Res..

[35] Leveque C., Mrakic Sposta S., Theunissen S., Germonpré P., Lambrechts K., Vezzoli A., Bosco G., Lévénez M., Lafère P., Guerrero F. (2023). Oxidative Stress Response Kinetics after 60 Minutes at Different (1.4 ATA and 2.5 ATA) Hyperbaric Hyperoxia Exposures. Int. J. Mol. Sci..

[36] Oh S., Lee E., Lee J., Lim Y., Kim J., Woo S. (2008). Comparison of the effects of 40% oxygen and two atmospheric absolute air pressure conditions on stress-induced premature senescence of normal human diploid fibroblasts. Cell Stress. Chaperones.

[37] Ciarlone G.E., Hinojo C.M., Stavitzski N.M., Dean J.B. (2019). CNS function and dysfunction during exposure to hyperbaric oxygen in operational and clinical settings. Redox Biol..

[38] Manning E.P. (2016). Central Nervous System Oxygen Toxicity and Hyperbaric Oxygen Seizures. Aerosp. Med. Hum. Perform..

[39] Kang T.S., Gorti G.K., Quan S.Y., Ho M., Koch R.J. (2004). Effect of hyperbaric oxygen on the growth factor profile of fibroblasts. Arch. Facial Plast. Surg..

[40] Huang X., Liang P., Jiang B., Zhang P., Yu W., Duan M., Guo L., Cui X., Huang M., Huang X. (2020). Hyperbaric oxygen potentiates diabetic wound healing by promoting fibroblast cell proliferation and endothelial cell angiogenesis. Life Sci..

[41] Hong W.X., Hu M.S., Esquivel M., Liang G.Y., Rennert R.C., McArdle A., Paik K.J., Duscher D., Gurtner G.C., Lorenz H.P. (2014). The Role of Hypoxia-Inducible Factor in Wound Healing. Adv. Wound Care.

[42] Lindenmann J., Kamolz L., Graier W., Smolle J., Smolle-Juettner F.M. (2022). Hyperbaric Oxygen Therapy and Tissue Regeneration: A Literature Survey. Biomedicines.

[43] Oley M.H., Oley M.C., Langi F., Langi Y.A., Keppel B.J., Tangkilisan A.N., Lampus H.F., Sipayung E.F., Aling D.M.R., Faruk M. (2021). Predicting hyperbaric oxygen therapy success using the decision tree approach. Ann. Med. Surg..

[44] Fosen K.M., Thom S.R. (2014). Hyperbaric oxygen, vasculogenic stem cells, and wound healing. Antioxid. Redox Signal.

[45] Boykin J.V., Baylis C. (2007). Hyperbaric oxygen therapy mediates increased nitric oxide production associated with wound healing: A preliminary study. Adv. Skin. Wound Care.

[46] Uusijärvi J., Eriksson K., Larsson A.C., Nihlén C., Schiffer T., Lindholm P., Weitzberg E. (2015). Effects of hyperbaric oxygen on nitric oxide generation in humans. Nitric Oxide.

[47] Růžička J., Dejmek J., Bolek L., Beneš J., Kuncová J. (2021). Hyperbaric oxygen influences chronic wound healing-a cellular level review. Physiol. Res..

[48] Susilo I., Devi A., Purwandhono A. (2022). The effects of hyperbaric oxygen therapy in improvement of Tnf- A, Hsp 70, Enos, Vegf, collagen 3, and Il 6 protein expression in wound healing. Int. J. Health Sci..

[49] Peña-Villalobos I., Casanova-Maldonado I., Lois P., Prieto C., Pizarro C., Lattus J., Osorio G., Palma V. (2018). Hyperbaric Oxygen Increases Stem Cell Proliferation, Angiogenesis and Wound-Healing Ability of WJ-MSCs in Diabetic Mice. Front. Physiol..

[50] O’Reilly D., Pasricha A., Campbell K., Burke N., Assasi N., Bowen J.M., Tarride J.E., Goeree R. (2013). Hyperbaric oxygen therapy for diabetic ulcers: Systematic review and meta-analysis. Int. J. Technol. Assess. Health Care.

[51] Löndahl M. (2013). Hyperbaric oxygen therapy as adjunctive treatment for diabetic foot ulcers. Int. J. Low. Extrem. Wounds.

[52] Sharma R., Sharma S.K., Mudgal S.K., Jelly P., Thakur K. (2021). Efficacy of hyperbaric oxygen therapy for diabetic foot ulcer, a systematic review and meta-analysis of controlled clinical trials. Sci. Rep..

[53] Brouwer R.J., Lalieu R.C., Hoencamp R., van Hulst R.A., Ubbink D.T. (2020). A systematic review and meta-analysis of hyperbaric oxygen therapy for diabetic foot ulcers with arterial insufficiency. J. Vasc. Surg..

[54] Savvidou O.D., Kaspiris A., Bolia I.K., Chloros G.D., Goumenos S.D., Papagelopoulos P.J., Tsiodras S. (2018). Effectiveness of Hyperbaric Oxygen Therapy for the Management of Chronic Osteomyelitis: A Systematic Review of the Literature. Orthopedics.

[55] Chen C.E., Ko J.Y., Fu T.H., Wang C.J. (2004). Results of chronic osteomyelitis of the femur treated with hyperbaric oxygen: A preliminary report. Chang Gung Med. J..

[56] Cooper J.S., Hanley M.E., Hendriksen S. (2025). Hyperbaric Treatment of Chronic Refractory Osteomyelitis. StatPearls.

[57] Shields R.C., Nichols F.C., Buchta W.G., Claus P.L. (2010). Hyperbaric oxygen therapy for chronic refractory osteomyelitis of the sternum. Ann. Thorac. Surg..

[58] Kjellberg A., Zhao A., Lussier A., Hassler A., Al-Ezerjawi S., Boström E., Catrina S.B., Bergman P., Rodriguez-Wallberg K.A., Lindholm P. (2024). Hyperbaric oxygen therapy as an immunomodulatory intervention in COVID-19-induced ARDS: Exploring clinical outcomes and transcriptomic signatures in a randomised controlled trial. Pulm. Pharmacol. Ther..

[59] Huang E.T., Mansouri J., Murad M.H., Joseph W.S., Strauss M.B., Tettelbach W., Worth E.R. (2015). A clinical practice guideline for the use of hyperbaric oxygen therapy in the treatment of diabetic foot ulcers. Undersea Hyperb. Med..

[60] Li W., Fu C., Xv L., Yang B., Liu G., Fan W. (2021). Hyperbaric oxygen therapy for chronic diabetic foot ulcers: An overview of systematic reviews. Diabetes Res. Clin. Pract..

[61] Zhang Z., Zhang W., Xu Y., Liu D. (2022). Efficacy of hyperbaric oxygen therapy for diabetic foot ulcers: An updated systematic review and meta-analysis. Asian J. Surg..

[62] Freiberger J.J., Padilla-Burgos R., Chhoeu A.H., Kraft K.H., Boneta O., Moon R.E., Piantadosi C.A. (2007). Hyperbaric oxygen treatment and bisphosphonate-induced osteonecrosis of the jaw: A case series. J. Oral Maxillofac. Surg..

[63] Shaw R., Butterworth C., Tesfaye B., Bickerstaff M., Dodd S., Smerdon G., Chauhan S., Brennan P., Webster K., McCaul J. (2018). HOPON (Hyperbaric Oxygen for the Prevention of Osteoradionecrosis): A randomised controlled trial of hyperbaric oxygen to prevent osteoradionecrosis of the irradiated mandible: Study protocol for a randomised controlled trial. Trials.

[64] Dholam K.P., Gurav S.V. (2012). Dental implants in irradiated jaws: A literature review. J. Cancer Res. Ther..

[65] Toneatti D.J., Graf R.R., Burkhard J.P., Schaller B. (2021). Survival of dental implants and occurrence of osteoradionecrosis in irradiated head and neck cancer patients: A systematic review and meta-analysis. Clin. Oral Investig..

[66] Chouinard A.F., Giasson L., Fortin M. (2016). Hyperbaric Oxygen Therapy for Head and Neck Irradiated Patients with Special Attention to Oral and Maxillofacial Treatments. J. Can. Dent. Assoc..

[67] Lin Z.C., Bennett M.H., Hawkins G.C., Azzopardi C.P., Feldmeier J., Smee R., Milross C. (2023). Hyperbaric oxygen therapy for late radiation tissue injury. Cochrane Database Syst. Rev..

[68] El-Rabbany M., Duchnay M., Raziee H.R., Zych M., Tenenbaum H., Shah P.S., Azarpazhooh A. (2019). Interventions for preventing osteoradionecrosis of the jaws in adults receiving head and neck radiotherapy. Cochrane Database Syst. Rev..

[69] Jenwitheesuk K., Mahakkanukrauh A., Punjaruk W., Jenwitheesuk K., Chowchuen B., Jinaporntham S., Uraiwan K., Limrattanapimpa P. (2018). Efficacy of Adjunctive Hyperbaric Oxygen Therapy in Osteoradionecrosis. Biores. Open Access.

[70] Mohandas R., Mohapatra S., Narkhede R., Kheur S. (2023). Effectiveness of Hyperbaric Oxygen Therapy in the Management of Osteoradionecrosis of the Jaw: A Systematic Review. J. Health Allied Sci. NU.

[71] Geçkil N. (2025). Treatment approaches in cases of mandibular osteoradionecrosis: A systematic meta-analysis. J. Stomatol. Oral Maxillofac. Surg..

[72] Fritz M.A., Arianpour K., Liu S.W., Lamarre E.D., Genther D.J., Ciolek P.J., Byrne P.J., Prendes B.L. (2025). Managing Mandibular Osteoradionecrosis. Otolaryngol. Head. Neck Surg..

[73] Dang B., Gamage S., Sethi S., Jensen E.D., Sambrook P., Goss A. (2023). The role of hyperbaric oxygen in osteoradionecrosis-a prophylactic insight. Aust. Dent. J..

[74] Peterson D.E., Koyfman S.A., Yarom N., Lynggaard C.D., Ismaila N., Forner L.E., Fuller C.D., Mowery Y.M., Murphy B.A., Watson E. (2024). Prevention and Management of Osteoradionecrosis in Patients with Head and Neck Cancer Treated with Radiation Therapy: ISOO-MASCC-ASCO Guideline. J. Clin. Oncol..

[75] He L., Sun X., Liu Z., Qiu Y., Niu Y. (2020). Pathogenesis and multidisciplinary management of medication-related osteonecrosis of the jaw. Int. J. Oral Sci..

[76] Ruggiero S.L., Dodson T.B., Aghaloo T., Carlson E.R., Ward B.B., Kademani D. (2022). American Association of Oral and Maxillofacial Surgeons’ Position Paper on Medication-Related Osteonecrosis of the Jaws-2022 Update. J. Oral Maxillofac. Surg..

[77] Freiberger J.J., Padilla-Burgos R., McGraw T., Suliman H.B., Kraft K.H., Stolp B.W., Moon R.E., Piantadosi C.A. (2012). What is the role of hyperbaric oxygen in the management of bisphosphonate-related osteonecrosis of the jaw: A randomized controlled trial of hyperbaric oxygen as an adjunct to surgery and antibiotics. J. Oral Maxillofac. Surg..

[78] Beth-Tasdogan N.H., Mayer B., Hussein H., Zolk O., Peter J.U. (2022). Interventions for managing medication-related osteonecrosis of the jaw. Cochrane Database Syst. Rev..

[79] Govaerts D., Piccart F., Ockerman A., Coropciuc R., Politis C., Jacobs R. (2020). Adjuvant therapies for MRONJ: A systematic review. Bone.

[80] Ceponis P., Keilman C., Guerry C., Freiberger J.J. (2017). Hyperbaric oxygen therapy and osteonecrosis. Oral Dis..

[81] Biancardi M., Júnior L., Fischer-Bullen I., Santos P. (2022). Hyperbaric Oxygen and Medication-Related Osteonecrosis of the Jaw (MRONJ): An Integrative Review. Int. J. Odontostomatol..

[82] Tanabe T., Kimura T., Sakata K.I., Ohga N., Yanagawa-Matsuda A., Kobori Y. (2025). Medication-Related Osteonecrosis Successfully Treated with Hyperbaric Oxygen Therapy and Conservative Treatment: A Case Report. Cureus.

[83] Frutuoso F., Freitas F., Vilares M., Francisco H., Marques D., Caramês J., Moreira A. (2024). Medication-Related Osteonecrosis of the Jaw: A Systematic Review of Case Reports and Case Series. Diseases.

[84] Byrne H., O’Reilly S., Weadick C.S., Brady P., Ríordáin R.N. (2024). How we manage medication-related osteonecrosis of the jaw. Eur. J. Med. Res..

[85] Liao J., Ren J., Qing W., Mu Y.D., Li P. (2020). Impact of Hyperbaric Oxygen on the Healing of Teeth Extraction Sockets and Alveolar Ridge Preservation. Clin. Oral Investig..

[86] Altug H.A., Tatli U., Coskun A.T., Erdogan Ö., Özkan A., Sencimen M., Kürkçü M. (2018). Effects of hyperbaric oxygen treatment on implant osseointegration in experimental diabetes mellitus. J. Appl. Oral Sci..

[87] Oliveira P.A., Oliveira A.M., Pablos A.B., Costa F.O., Silva G.A., Santos J.N., Cury P.R. (2012). Influence of hyperbaric oxygen therapy on peri-implant bone healing in rats with alloxan-induced diabetes. J. Clin. Periodontol..

[88] Chambrone L., Mandia J., Shibli J.A., Romito G.A., Abrahao M. (2013). Dental implants installed in irradiated jaws: A systematic review. J. Dent. Res..

[89] Esposito M., Worthington H.V. (2013). Interventions for replacing missing teeth: Hyperbaric oxygen therapy for irradiated patients who require dental implants. Cochrane Database Syst. Rev..

[90] Benites Condezo A.F., Araujo R.Z., Koga D.H., Curi M.M., Cardoso C.L. (2021). Hyperbaric oxygen therapy for the placement of dental implants in irradiated patients: Systematic review and meta-analysis. Br. J. Oral Maxillofac. Surg..

[91] Cho Y.D., Kim K.H., Lee Y.M., Ku Y., Seol Y.J. (2021). Periodontal Wound Healing and Tissue Regeneration: A Narrative Review. Pharmaceuticals.

[92] Chen T.-L., Xu B., Liu J.-C., Li S.-G., Li D.-Y., Gong G.-C., Wu Z.-F., Lin S.-L., Zhou Y.-J. (2012). Effects of hyperbaric oxygen on aggressive periodontitis and subgingival anaerobes in Chinese patients. J. Indian Soc. Periodontol..

[93] Burcea A., Mihai L.L., Bechir A., Suciu M., Bechir E.S. (2022). Clinical Assessment of the Hyperbaric Oxygen Therapy Efficacy in Mild to Moderate Periodontal Affections: A Simple Randomised Trial. Medicina.

[94] Latusek K., Słotwińska-Pawlaczyk A., Warakomska A., Kubicka-Musiał M., Wiench R., Orzechowska-Wylęgała B. (2023). Pilot Study: The Effectiveness of Hyperbaric Oxygen Therapy in the Treatment of Periodontitis in Patients with Type 2 Diabetes. Healthcare.

[95] Nogueira-Filho G.R., Rosa B.T., David-Neto J.R. (2010). Effects of hyperbaric oxygen therapy on the treatment of severe cases of periodontitis. Undersea Hyperb. Med..

[96] Lombardo G., Pardo A., Signoretto C., Signoriello A., Simeoni E., Rovera A., Nocini P.F. (2020). Hyperbaric oxygen therapy for the treatment of moderate to severe periodontitis: A clinical pilot study. Undersea Hyperb. Med..

[97] Giacon T.A., Giancola F., Paganini M., Tiengo C., Camporesi E.M., Bosco G. (2021). Hyperbaric Oxygen Therapy and A-PRF Pre-Treated Implants in Severe Periodontitis: A Case Report. Int. J. Environ. Res. Public Health.

[98] Heck T., Lohana D., Mallela D., Mandil O., Sun L., Saxena P., Decker A.M., Wang H.-L. (2024). Hyperbaric oxygen therapy as an adjunct treatment of periodontitis, MRONJ, and ONJ: A systematic literature review. Clin. Oral Investig..

[99] Winsness A., Fallin G., Herring K. (2024). What is the Effect of Hyperbaric Oxygen Therapy on Pocket Depths as an Adjunct to Conventional Scaling and Root Planning?. Dent. Rev..

[100] Berglund C., Ekströmer K., Abtahi J. (2015). Primary Chronic Osteomyelitis of the Jaws in Children: An Update on Pathophysiology, Radiological Findings, Treatment Strategies, and Prospective Analysis of Two Cases. Case Rep. Dent..

[101] García C.M., García M.A., García R.G., Gil F.M. (2013). Osteomyelitis of the mandible in a patient with osteopetrosis. Case report and review of the literature. J. Maxillofac. Oral Surg..

[102] Lentrodt S., Lentrodt J., Kübler N., Mödder U. (2007). Hyperbaric oxygen for adjuvant therapy for chronically recurrent mandibular osteomyelitis in childhood and adolescence. J. Oral Maxillofac. Surg..

[103] Stark Z., Savarirayan R. (2009). Osteopetrosis. Orphanet J. Rare Dis..

[104] Murai C., Sakata K.-i., Nakamura K., Yoshikawa K., Sato J., Matsuda A., Kitagawa Y. (2023). Refractory maxillary osteomyelitis with osteopetrosis: A case report. J. Oral Maxillofac. Surg. Med. Pathol..

[105] Sharma A., Ingole S.N., Deshpande M.D., Kazi N., Meshram D., Ranadive P. (2020). A Rare Case of Osteoclast-poor Osteopetrosis (RANKL Mutation) with Recurrent Osteomyelitis of Mandible: A Case Report. Int. J. Clin. Pediatr. Dent..

[106] Monis P.L., Bhat V., Shetty S., Salins P.C. (2021). Hyperbaric Oxygen Therapy in the Management of Zygomatic Bone Osteomyelitis. J. Maxillofac. Oral Surg..

[107] Kato C., de Arruda J.A.A., Mendes P.A., Neiva I.M., Abreu L.G., Moreno A., Silva T.A., Souza L.N., Mesquita R.A. (2020). Infected Cemento-Osseous Dysplasia: Analysis of 66 Cases and Literature Review. Head. Neck Pathol..

[108] Kumar Y., Anand R., Bhagat N., Chakarvarty K., Jaiswal Y. (2024). Florid Cemento-Osseous Dysplasia Presenting as Secondary Osteomyelitis: A Case Report of an Undiagnosed Condition. Cureus.

[109] Bokhari S.F.H., Bakht D., Amir M., Haris H.M., Ain N.U., Qureshi M.S., Yousaf F., Yousaf R., Ali K., Javed M.A. (2025). Granulicatella infections: Comprehensive review of an elusive opportunistic pathogen. World J. Clin. Cases.

[110] Gawdi R., Yrastorza J., Cooper J.S. (2025). Hyperbaric Oxygen Therapy Contraindications. StatPearls.

[111] Baude J., Cooper J.S. (2025). Hyperbaric Contraindicated Chemotherapeutic Agents. StatPearls.

[112] Camporesi E.M. (2014). Side effects of hyperbaric oxygen therapy. Undersea Hyperb. Med..

[113] Torp K.D., Carraway M.S., Ott M.C., Stolp B.W., Moon R.E., Piantadosi C.A., Freiberger J.J. (2012). Safe administration of hyperbaric oxygen after bleomycin: A case series of 15 patients. Undersea Hyperb. Med..

[114] Sadri R.A., Cooper J.S. (2025). Hyperbaric Complications. StatPearls.

[115] Karagoz B., Suleymanoglu S., Uzun G., Bilgi O., Aydinoz S., Haholu A., Turken O., Onem Y., Kandemir E.G. (2008). Hyperbaric oxygen therapy does not potentiate doxorubicin-induced cardiotoxicity in rats. Basic. Clin. Pharmacol. Toxicol..

[116] Brenna C.T., Khan S., Djaiani G., Buckey J.C., Katznelson R. (2022). The role of routine pulmonary imaging before hyperbaric oxygen treatment. Diving Hyperb. Med..

[117] Brenna C.T.A., Khan S., Djaiani G., Au D., Schiavo S., Wahaj M., Janisse R., Katznelson R. (2023). Pulmonary function following hyperbaric oxygen therapy: A longitudinal observational study. PLoS ONE.

[118] DuBose K.J., Cooper J.S. (2025). Hyperbaric Patient Selection. StatPearls.

[119] Lee J.-H., Lee H.-Y., Sun K.-H., Heo T., Lee S.-M. (2025). Risk Factors for Middle Ear Barotrauma in Patients with Carbon Monoxide Poisoning Undergoing Monoplace Hyperbaric Oxygen Therapy: A Retrospective Cohort Study. J. Clin. Med..

[120] Lima M.A., Farage L., Cury M.C., Bahmad F. (2012). Middle ear barotrauma after hyperbaric oxygen therapy-the role of insuflation maneuvers. Int. Tinnitus J..

[121] Park S., Marinov A., Clarke H., Schiavo S., Greer E., Djaiani G., Tarshis J., Katznelson R. (2025). Safety of hyperbaric oxygen therapy in non-emergent patients with a history of seizures: A retrospective cohort study. PLoS ONE.

[122] Schiavo S., Brenna C.T.A., Albertini L., Djaiani G., Marinov A., Katznelson R. (2024). Safety of hyperbaric oxygen therapy in patients with heart failure: A retrospective cohort study. PLoS ONE.

